# Calibrating Seed-Based Heuristics to Map Short Reads With Sesame

**DOI:** 10.3389/fgene.2020.00572

**Published:** 2020-06-25

**Authors:** Guillaume J. Filion, Ruggero Cortini, Eduard Zorita

**Affiliations:** ^1^Center for Genomic Regulation (CRG), The Barcelona Institute of Science and Technology, Barcelona, Spain; ^2^University Pompeu Fabra (UPF), Barcelona, Spain

**Keywords:** C library, probability, analytic combinatorics, seeding accuracy, heuristic algorithms

## Abstract

The increasing throughput of DNA sequencing technologies creates a need for faster algorithms. The fate of most reads is to be mapped to a reference sequence, typically a genome. Modern mappers rely on heuristics to gain speed at a reasonable cost for accuracy. In the seeding heuristic, short matches between the reads and the genome are used to narrow the search to a set of candidate locations. Several seeding variants used in modern mappers show good empirical performance but they are difficult to calibrate or to optimize for lack of theoretical results. Here we develop a theory to estimate the probability that the correct location of a read is filtered out during seeding, resulting in mapping errors. We describe the properties of simple exact seeds, skip seeds and MEM seeds (Maximal Exact Match seeds). The main innovation of this work is to use concepts from analytic combinatorics to represent reads as abstract sequences, and to specify their generative function to estimate the probabilities of interest. We provide several algorithms, which together give a workable solution for the problem of calibrating seeding heuristics for short reads. We also provide a C implementation of these algorithms in a library called Sesame. These results can improve current mapping algorithms and lay the foundation of a general strategy to tackle sequence alignment problems. The Sesame library is open source and available for download at https://github.com/gui11aume/sesame.

## 1. Introduction

### 1.1. Mapping Reads to Genomes

Say we use an imperfect instrument to sequence a small fragment of DNA. If we know its genome of origin, how can we find the location of the fragment in this genome?

The advent of high-throughput sequencing has put this question at the center of countless applications in genetics, such as discovering disease-causing mutations, selecting breeds of interest in agriculture or tracing human migrations.

We will refer to this as the *true location problem*. The answer to the problem depends on the length of the read, on the error rate of the sequencer and on one key feature of the genome: its repeat structure. Most genomes contain repetitive sequences, i.e., relatively large subsequences that are present at multiple locations. If the sequenced DNA fragment comes from a repetitive sequence, it may be impossible to map the read with certainty.

In general, repetitive sequences are not identical but merely homologous—meaning that their similarity is very unlikely to occur by chance. So if the sequenced DNA fragment originates from a repetitive sequence, we can map the read only if we can rule out all the candidates except one. This, in turn, depends on the similarity between the duplicates and on the error rate of the sequencer.

Since repetitive sequences play a central role in the problem, we give the terms “targets” and “duplicates” a meaning that will facilitate the exposition of the theory.

**Definition 1**. *The target is the DNA fragment that was actually sequenced. Duplicates are sequences of the genome that share homology with the target (in genetics they are often referred to as paralogs). In this article we will focus on short reads from complex eukaryotic genomes, so for concreteness the reader can assume that fragments are 30–300 bp long and that duplicates have above 75% identity with the target*.

The difficulty of the true location problem is due to sequencing errors. Occasionally, the sequence of a DNA fragment can be closer to one of the duplicates than to the target. So the *true* location of a DNA fragment is not always the *best* (as measured by the identity between the sequence and the candidate location). This naturally leads to asking how we can identify the *best* location of the fragment in the genome.

We will refer to this as the *best location problem*. It amounts to finding the optimal alignment between two sequences, and for this reason has received substantial attention in bioinformatics. For the purpose of developing a theory rooted in statistics, our main concern is to address the true location problem, but for simplicity, we will assume that the true location is also the best. When applicable, we will clarify the implications of this hypothesis in the relevant sections, and we will see how it impacts the theory as a whole in section 8.1.

### 1.2. Seeding Heuristics

Exact alignment algorithms were designed to solve the best location problem (Needleman and Wunsch, 1970; Smith and Waterman, 1981), but they are too slow to process the large amount of data generated by modern sequencers. Instead, one uses heuristic methods, i.e., algorithms that run faster but may return an incorrect result (Waterman, 1984).

The most popular heuristic for mapping DNA sequences is a filtration method called “seed-and-extend.” The principle is to first identify seeds, defined as short regions of high similarity between the read and the genome, and then use an exact alignment algorithm at the seeded locations to evaluate the candidates and identify the optimum. Exact alignment algorithms, such as the Needleman-Wunsch (Needleman and Wunsch, 1970) or the Smith-Waterman (Smith and Waterman, 1981) algorithms return the distance or the similarity between two sequences according to some quantitative criterion. They are exact in the sense that they always return the correct answer, unlike heuristic algorithms. The seed-and-extend strategy was first proposed in FASTA (Lipman and Pearson, 1985) and BLAST (Altschul et al., 1990) to search for homology in sequence databases of proteins and DNA.

There are efficient methods to extract seeds, so it is possible to quickly hone in on a small set of candidates and to reduce the search space of the alignment algorithm. The disadvantage is that the target may not be in the candidate set, in which case the read cannot be mapped correctly.

As a consequence, seeding methods induce a trade-off between speed and accuracy: If the filtration is set to produce a large candidate set, the target is likely to be discovered, with the downside that checking all the candidates with an exact alignment algorithm will take long. Conversely, if the filtration is set to produce a small candidate set, the process will run faster but the target is more likely to be missed.

Importantly, the trade-off depends on the seeding method. This means that mapping algorithms can run faster at no cost for accuracy if we can find better seeding strategies. Progress on this line of research has largely benefited from the improvement of computer hardware and from the development of optimized data structures. There already exists a large body of literature on the design of seeding algorithms; the interested reader can find examples of those in references (Ma et al., 2002; Brejová, 2003; Li et al., 2004; Kucherov et al., 2005; Sun and Buhler, 2005; Xu et al., 2006; Lin et al., 2008). Sun and Buhler (2006) and Li and Homer (2010) present high-level comparisons of different designs, and Navarro (2001) gives a global overview of filtration methods in pattern matching.

### 1.3. The Two Types of Seeding Failure

Filtering heuristics are considered to fail whenever the target is not in the candidate set, but here we must be more specific and distinguish two kinds of failure: In the first kind, the candidate set contains a duplicate of the target but does not contain the target itself; in the second kind, the candidate set contains neither the target nor any duplicate.

The distinction is important because the duplicates of the target are similar to the read (due to their similarity to the target), so a failure of the first kind often looks like a success. In contrast, a failure of the second kind is easier to flag because in this case the candidates are not similar to the read. We will simply assume that seeding failures of the second kind are always detected (as explained in section 8.3), so that we can focus on the more difficult case of seeding failures of the first kind.

Before going further, we introduce three terms that will simplify the exposition.

**Definition 2**. *The output of the seeding step is the candidate set. The candidate set is the list of genomic locations where the read can be potentially mapped. The read is always mapped to one element of the candidate set. The seeding step is said to be*

- “on-target” if the candidate set contains the target,
- “off-target” if the candidate set contains a duplicate but not the target,
- “null” if the candidate set contains neither.

*In this article, we will always consider that a genomic location is in the candidate set if and only if the read contains at least one seed with a perfect match for this genomic location*.

With our assumptions, a read is mapped to the wrong location if and only if the candidate set is off-target. Indeed, if the candidate set is null, the read is not mapped, and if the candidate set is on-target, the correct location is discovered at the alignment step. The equivalence is granted by the assumption that the true location is also the best, reducing the mapping problem to a seeding problem. That being said, the true location is not always the best in practical applications. We will show in section 8.1 that off-target seeding can be responsible for most of the mapping errors even without the assumption above, but for now we maintain the strict equivalence between mapping errors and off-target seeding.

Focusing on popular heuristics to map short reads, our aim is to develop a method to estimate the probability that the candidate set is off-target. Previous work pioneered a method to compute seeding probabilities but it did not distinguish off-target from null seeding (Filion, 2017, 2018), and therefore did not provide a way to estimate the frequency of mapping errors. Other authors investigated the reliability of mapping algorithms (Menzel et al., 2013), but they focused on the probabilities of random hits, recognizing that addressing the problem of incorrect mapping requires taking into consideration the repeat structure of the genome.

The rest of the article is organized as follows: section 2 presents common seeding strategies used in bioinformatics, section 3 presents the basic concepts of analytic combinatorics that will be required to compute seeding probabilities, sections 4 to 6 treat the cases of exact seeds, skip seeds and MEM seeds, three common seeding heuristic used for mapping, section 7 presents Sesame, a C library implementing the main results of the theory, section 8 returns to the mapping problem and revisits the assumptions of the model, and finally section 9 provides some perspectives on the present work. Appendix A gathers for reference all the definitions encountered in the text, and Appendix B contains proofs and complements omitted from the main text.

## 2. Seeds

The term “seed” has different meanings in computational biology. It can designate a part of the read, a part of the genome, a particular sequence motif, or a structured pattern of matches. Also, a seed does not always refer to an exact match. For instance, the algorithm PatternHunter (Ma et al., 2002) uses spaced seeds that tolerate mismatches. To avoid any confusion, we will adopt the convention below.

**Definition 3**. *A seed is a subsequence of the read that has size at least γ (defined by the context of the problem) and that has at least one perfect match in the reference genome. Every genomic match of every seed is in the candidate set*.

When a seed matches a given location of the genome, we say that it is a seed for that location. This is particularly useful in expressions such as “seed for the target” or “seed for a duplicate.”

This definition presents a computational challenge: to know if a given subsequence of a read is a seed we need to know if it exists somewhere in the genome. This is a non trivial problem in itself, but fortunately we can use practical methods to solve it, even when the reference genome is very large.

These algorithms are crucial for the present theory, but describing them in depth is outside the scope of this document. Let us just mention that all the methods belong to a family known as exact offline string matching algorithms, where “offline” means that sequences are looked up in an index instead of the genome itself. Online methods can be used when the reference genome is not indexed (Faro and Lecroq, 2013), but this case is of little relevance in the present context.

The index is usually a hash table or a variant of the so-called FM-index (Ferragina and Manzini, 2000, 2005). Hash tables are typically used to index *k*-mers, which makes them useful to search for seeds of fixed size *k* (see Manekar and Sathe, 2018 for a recent benchmark of *k*-mer hashing algorithms). In contrast, some text-indexing structures have no set size so they can be used to search for seeds of different lengths. This is the case of the FM-index, a compact data structure based on the suffix array (Manber and Myers, 1993) and on the Burrows-Wheeler transform (Burrows and Wheeler, 1994), emulating a suffix trie with a much smaller memory footprint (Ferragina and Manzini, 2000, 2005).

Other methods can be efficient (e.g., running a bisection on the suffix array Dobin et al., 2013) but the FM-index is currently the most popular choice for seeding methods. For MEM seeds defined below, it is even the only practical option (Khan et al., 2009; Vyverman et al., 2013; Fernandes and Freitas, 2014; Khiste and Ilie, 2015). Overall, the detail is of little interest for the theory. We simply assume that seeds are known at all times without ambiguity because this problem has several practical solutions.

### 2.1. Exact Seeds

Exact seeds are seeds of fixed size γ. In other words, when using exact seeds, the candidate set consists of all the genomic locations for which there is a perfect match of size γ in the read. This seeding heuristic was used in the first version of BLASTN (Altschul et al., 1990), but it has become unpopular for producing many short spurious hits.

**Figure 1:**
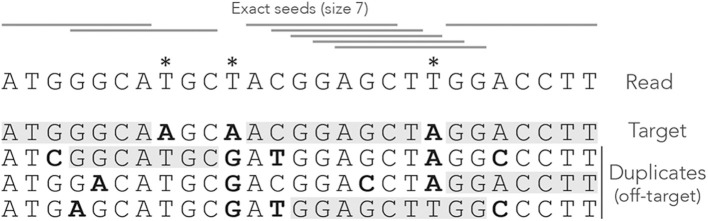
Exact seeding. A sequenced DNA fragment (Read) is shown above the actual molecule (Target), and above three paralogs of the target (Duplicates). Sequencing errors are indicated by 
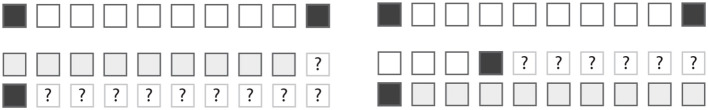
 (all three are A misread as T). The mismatches between the genomic sequences and the read are indicated in bold. The exact seeds of size γ = 7 are indicated as horizontal gray lines above the read. Matching regions in the genomic sequences are shadowed in gray. Several overlapping seeds accumulate at the center of the read, which is typical of exact seeds.

Figure 1 shows the exact seeds from an example read with three miscalled nucleotides. The sequenced DNA fragment has three duplicates so the seeds can match four possible locations.

Observe that erroneous nucleotides can belong to exact seeds because they sometimes match a duplicate. For instance, the first sequencing error matches all the duplicates and belongs to an off-target seed for the first duplicate. However, sequencing errors that are mismatches for *all* the sequences cannot belong to a seed. This is the case of the second sequencing error in the example, creating a local deficit of seeds.

Note the clutter in the middle of the read, where consecutive seeds match consecutive sequences at the same location. This is typical for exact seeds and is considered a nuisance for the implementation. Indeed, it is a waste of computer resources to discover matches in sequences that are already in the candidate set. In addition, this seeding method is not particularly sensitive compared to spaced seeds (Ma et al., 2002) so it is used only in a few specific applications. Nevertheless, it will be useful for the development of the present theory.

### 2.2. Skip Seeds

Skip seeds have a fixed size γ, but unlike exact seeds they cannot start at every nucleotide. Instead, a certain amount of nucleotides is skipped between every seed. This is a way to reduce the overlapping matches at the same location, at the cost of sensitivity. This seeding heuristic is the core of Bowtie2 (Langmead and Salzberg, 2012), where seeds have size γ = 16 and are separated by 10 nucleotides (nine positions are skipped). We will refer to seeds where *n* nucleotides are skipped as “skip-*n* seeds.” For instance, Bowtie2 uses skip-9 seeds.

**Figure 2:**
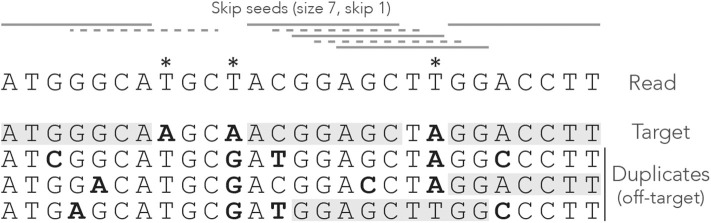
Example of skip seeds. The sequences and the annotations are the same as in Figure 1, but here we use skip-1 seeds. In other words, seeds can never start at nucleotides 2, 4, 6 etc. To highlight the difference with Figure 1, the missing seeds are represented by dotted lines.

Figure 2 shows what happens when exact seeds are replaced by skip-1 seeds on the read of Figure 1. Here the size is still γ = 7 but 1 nucleotide is skipped between seeds. This amounts to removing every second seed. The consequence is that there are fewer overlapping matches at the center of the read, but the only seed for the second duplicate is lost. This is a rather positive outcome because there is one off-target location fewer in the candidate set, but the same might happen to the target.

It is clear that skipping nucleotides reduces the sensitivity of the seeding step, but to what extent? One could test this empirically, but the answer depends on the seed length, the number of nucleotides that are skipped, the error rate of the sequencer and the size of the reads. The present theory will allow us to make general statements about the performance of skip seeds against exact seeds in different contexts.

### 2.3. MEM Seeds

MEM seeds (where MEM stands for Maximal Exact Match) are somewhat harder to define. Unlike exact seeds and skip seeds, their size is variable. They are used in BWA-MEM (Li, 2013) where they give good empirical results. To describe MEM seeds, let us first clarify the meaning of “Maximal Exact Match.”

**Definition 4**. *A Maximal Exact Match (MEM) is a subsequence of the read that is present in the reference genome and that cannot be extended—either because the read ends or because the extended subsequence is not in the genome*.

**Figure 3:**
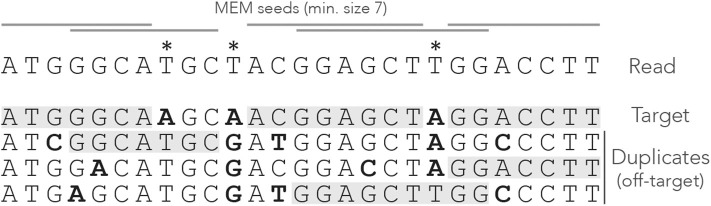
Example of MEM seeds. The sequences and the annotations are the same as in Figure 1, but here we use MEM seeds of minimum size γ = 7. The clutter at the center of the read has disappeared and there is at least one seed for each sequence.

A *MEM seed* is simply a MEM of size γ or greater. Figure 3 shows what happens when using MEM seeds on the read of Figure 1. Observe that the clutter at the center of the read has disappeared because consecutive matches are fused into a few MEM seeds.

Two consecutive MEM seeds can overlap, in which case they always match distinct sequences of the genome (otherwise neither of them would be a MEM seed). There does not have to be any overlap though, because a nucleotide can be a mismatch against *all* the sequences, like the second read error for instance.

Note that a MEM does not always match a single subsequence of the genome. For instance, the rightmost MEM seed matches two distinct genomic subsequences. This case motivates the following definition, which will play an important role later.

**Definition 5**. *A strict MEM seed has a single match in the genome. A *shared* MEM seed has several matches in the genome*.

**Figure 4:**
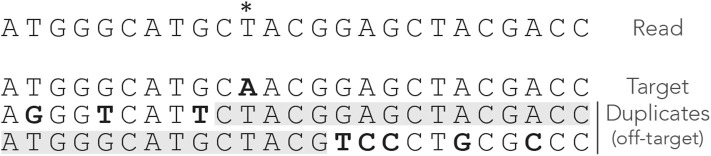
Issues with MEM seeds: inaccessible targets. The read, the MEM seeds and the sequences are represented as in Figure 3. The MEM seeds matching the two duplicates at the bottom effectively hide the target, so it cannot be discovered. This can occur even when the true location is the best candidate and when there is a single error on the read.

Compared to seeds of fixed size, MEM seeds have two counter-intuitive properties. The first is that there are cases where there cannot be any on-target seed, even when changing the minimum seed size γ. Figure 4 shows such an example. Even though there is a single sequencing error, the read has no MEM seed for the target. Lowering γ does not change this, so there is no way to discover the true location using MEM seeds (even though it is the best location).

**Figure 5:**
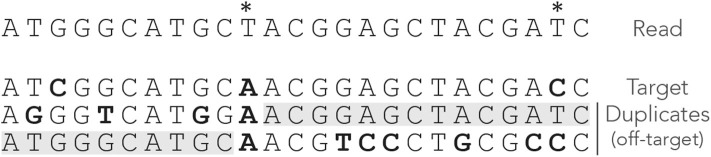
Issues with MEM seeds: too long reads. The read, the MEM seeds, and the sequences are represented as in Figure 3. There would be an on-target seed (shared) if the read were two nucleotides shorter. The true location is hidden by the last two nucleotides.

The second counter-intuitive property is that shortening a read can sometimes generate a MEM seed for the target. Figure 5 shows an example of this case. There is no MEM seed for the target, but there would be if the read were two nucleotides shorter on the right side. Indeed, in this case there would be a shared MEM seed matching the target and the first duplicate (provided γ ≤ 12).

These examples show that MEM seeds can perform worse than seeds of fixed size. MEM seeds yield fewer candidates and therefore speed up the mapping process, but the question is at what cost? The theory developed here will allow us to compute the probability that a read has no MEM seed for the target and thus that the true location is missed at the seeding stage.

### 2.4. Spaced Seeds

Originally introduced by Califano and Rigoutsos (1993) and popularized by PatternHunter (Ma et al., 2002), spaced seeds feature “don't-care” positions allowing them to detect imperfect matches. Spaced seeds are represented by models such as “11*11,” here indicating that the seeds have length 5 and that the middle position is disregarded. At index time, the genome is scanned with the model so that nucleotides labeled “1” are concatenated and indexed. At search time, the query is scanned with the model and the concatenated nucleotides labeled “1” are looked up in the index.

Spaced seeds of weight *w* (the number of 1 in the model) have the same memory requirements as contiguous seeds of length *w* but they are more sensitive (Li et al., 2006), making them very attractive for homology search. They were used in the first generation of short-read mappers (Jocham et al., 1986; Lin et al., 2008; Chen et al., 2009; Rumble et al., 2009), but nowadays they find more applications in genome comparisons (Kiełbasa et al., 2011; Healy, 2016), metagenomics (Břinda et al., 2015; Ounit and Lonardi, 2016), genome assembly (Birol et al., 2015), and long-read mapping (Sovic et al., 2016).

The success of the mainstream mappers BWA-MEM (Li, 2013) and Bowtie2 (Langmead and Salzberg, 2012) is due in part to the FM-index, which only supports contiguous seeds. Some workarounds are available for spaced seeds (Horton et al., 2008; Gagie et al., 2017) but they increase the memory footprint, explaining that short reads are typically mapped using contiguous seeds. More generally, computing the sensitivity of spaced seeds is challenging (Kucherov et al., 2006; Li et al., 2006; Martin and Noé, 2017). It is possible to do this using the tools introduced below, as shown in section 3.5. However, to compute off-target probabilities, the main purpose of this article, the complexity rapidly becomes prohibitive. We will thus restrict our attention to contiguous seeds because they are relevant for mapping problems and fit tightly within the present theory.

## 3. Model and Strategy

### 3.1. Sequencing Errors and Divergence of the Duplicates

We now need to model the sequencing and duplication processes so that we can compute the probabilities of the events of interest. We assume that the sequencing instrument has a constant substitution rate *p*, and that insertions and deletions never occur. When a substitution occurs, we assume that the instrument is equally likely to output any one of the remaining three nucleotides. This corresponds more or less to the error spectrum of the Illumina sequencing technology (Nakamura et al., 2011).

Next, we assume that the target sequence has *N* ≥ 0 duplicates, so that there are *N* off-target sequences. We further assume that duplication happened instantaneously at some point in the past and that all *N* + 1 sequences diverge independently of each other at a constant rate. In other words, we ignore the complications due to the genealogy of the duplication events. Instead, we simply assume that at each nucleotide position, any given duplicate is identical to the target with probability 1 − μ. If it is not, we assume that the duplicate sequence can be any of one the three remaining nucleotides (i.e., each is found with probability μ/3).

Note that read errors are always mismatches against the target (because we assume that the target is the true sequence), and they match each duplicate with probability μ/3. Correct nucleotides are always matches for the target, and they match each duplicate with probability 1 − μ.

Before going further, we also need to move a practical consideration out of the way. Seeds can match any sequence of the genome, not just the target or the duplicates. However, we will ignore matches in the rest of the genome because such random matches are unlikely to cause a mapping error when seeding is off-target, contrary to matches in duplicates. Neglecting those will greatly simplify the exposition of the theory without loss of generality. We will explain in section 8.3 how to deal with this practical case and how to identify those matches as off-target. Until then, we will consider that the target and the duplicates are the only sequences in the reference genome.

### 3.2. Weighted Generating Functions

The central object of analytic combinatorics is the generating function, and for our purpose we will use a special kind known as *weighted generating function*.

**Definition 6**. *Let* A *be a set of combinatorial objects such that* a∈A *has a size* |*a*| ∈ ℕ *and a weight w*(*a*) ∈ ℝ^+^. *The weighted generating function of* A *is defined as*

(1) $$A(z)=\sum_{a\in A}w(a)z^{\left|a\right|},$$

*Expression (1) also defines a sequence* (*a*_*k*_)_*k*≥0_ *such that*

$$A(z)=\sum_{k=0}^{\infty }a_{k}z^{k}.$$

*By definition* $a_{k}=\underset{a\in A_{k}}{\sum }w\left(a\right)$, *where A*_*k*_ *is the class of objects of size k in* A. *The number a*_*k*_ *is the total weight of objects of size k*.

To give an example, assume that a particular symbol, say ⇓, has a probability of occurrence equal to *p*. The weighted generating function of words containing only this symbol is *pz*. The weight of the word is its probability (here equal to *p*) and the size is its length (here 1).

In this document we will focus on the weighted generating function *A*(*z*) of the set A of reads that do not have on-target seeds (i.e., reads for which seeding is either null or off-target). The weight of a read is its probability of occurrence and the size *k* is its number of nucleotides. The coefficient *a*_*k*_ is thus the proportion of reads of size *k* that do not have an on-target seed, which is the quantity of interest.

The motivation for introducing weighted generating functions is that operations on combinatorial objects translate into operations on their weighted generating functions. If *A*(*z*) and *B*(*z*) are the weighted generating functions of two mutually exclusive sets A and B, the weighted generating function of A∪B is *A*(*z*) + *B*(*z*), as evident from expression (1). Size and weight can be defined for pairs of objects in A×B as |(*a, b*)| = |*a*| + |*b*| and *w*(*a, b*) = *w*(*a*)*w*(*b*). In other words the sizes are added and the weights are multiplied. With this convention, the weighted generating function of the Cartesian product A×B is *A*(*z*)*B*(*z*). This simply follows from expression (1) and from

$$A(z)B(z)=\sum_{a\in A}w(a)z^{\left|a\right|}\sum_{b\in ℬ}w(b)z^{\left|b\right|}=\text{​​}\sum_{(a,b)\in A\times ℬ}w(a)w(b)z^{\left|a|+|b\right|}.$$

These two operations are all we need in order to compute the weighted generating functions of the reads of interest. Addition corresponds to creating a new family by merging reads from families A and B. Multiplication corresponds to creating a new family by concatenating reads from families A and B.

### 3.3. Analytic Representation

The analytic combinatorics framework relies on a strategy referred to as the *symbolic method* (Sedgewick and Flajolet, 2013). The idea is to combine simple objects into more complex objects. Each combinatorial operation on the objects corresponds to a mathematical operation on their weighted generating functions. One can thus obtain the weighted generating function of complex objects, whose coefficients *a*_*k*_ (*k* ≥ 0) are the probabilities of interest (Régnier, 2000; Nicodeme et al., 2002; Sedgewick and Flajolet, 2013).

As explained by Filion (2017, 2018), we recode the reads in alphabets of custom symbols and we specify a construction plan of the reads using a so-called *transfer matrix* *M*(*z*). The transfer matrix specifies which types of “segments” can follow each other in the reads of interest: the entry at coordinates (*i, j*) is the weighted generating function of segments of type *j* that can be appended to segments of type *i*.

*M*(*z*) contains the weighted generating functions of all the reads that consist of a single segment. From the basic operations on weighted generating functions, *M*(*z*)^*s*^ contains the weighted generating functions of all the reads that consist of *s* segments. Therefore, the entry at coordinates (*i, j*) of the matrix *M*(*z*) + *M*(*z*)^2^ + *M*(*z*)^3^ + … = *M*(*z*) · (*I* − *M*(*z*))^−1^ is the weighted generating function of the reads of any size and any number of segments, that end with a segment of type *j* and that can be appended to a segment of type *i*. The examples below will clarify the key steps of this strategy.

A complete description of how to compute seeding probabilities with the symbolic method is given by Filion (2017, 2018). The interested readers can also find more about analytic combinatorics in popular textbooks (Flajolet and Sedgewick, 2009; Sedgewick and Flajolet, 2013).

### 3.4. Example 1: On-Target Exact Seeds

We highlight the strategy above with an example that will turn out to be central for the development of the theory. In addition, it is simple enough to provide a gentle introduction to the general methodology. This example was described in detail by Filion (2017, 2018), but we repeat it here with a different formalism to fit the present article.

The first step is to note that the nucleotide sequence of the read is irrelevant. Indeed, the read has an on-target seed if and only if it contains a stretch of γ nucleotides without error. For this reason, we recode reads as sequences of correct or erroneous nucleotides.

We define the mismatch alphabet $A_{0}=\left\{□,|,⇓\right\}$, where □ represents a correct nucleotide, ⇓ represents an erroneous nucleotide and | is a special symbol appended after the last nucleotide to mark the end of the read. It is not associated with a nucleotide and therefore has size 0.

This recoding allows us to partition the read in an important way.

**Definition 7**. *A terminator is any symbol that is different from the symbol* □. A *segment is a sequence of 0 or more* □ *symbols followed by a terminator. The tail is the last segment of the read, where the terminator is always the special symbol* |.

**Figure 6:**
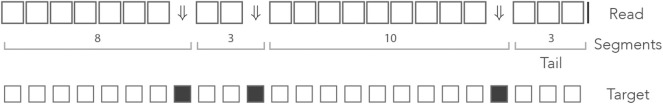
The mismatch encoding. An example read is represented in the mismatch alphabet. The symbol ⇓ represents a mismatch against the target (an erroneous nucleotide) and the symbol □ represents a match (a correct nucleotide). The symbol | is appended to the end of the read. The symbolic sequence of the target is represented below, where an open square stands for a match and a closed square stands for a mismatch.

Since the decomposition of the read in segments is unique, we can view a read as a sequence of segments with a tail, instead of a sequence of nucleotides. Figure 6 shows an example of decomposition in segments. On-target seeds cannot contain sequencing errors, therefore they must be completely embedded in a segment. So the sizes of the segments indicate whether the read contains an on-target seed or not.

The probability of occurrence of the symbol ⇓ is *p* (the error rate of the sequencer) so the probability of occurrence of the □ symbol is 1 − *p* = *q*. Both symbols have size 1, so their respective weighted generating functions are *pz* and *qz*. Using the rule for concatenation, we see that a segment of *i* symbols □ followed by a terminator has weighted generating function (*qz*)^*i*^*pz*. The final symbol | has size 0, so a tail segment of *i* symbols □ followed by the symbol | has weighted generating function (*qz*)^*i*^.

The key insight is that the reads without on-target seed are exactly the reads that are made of segments with fewer than γ symbols □, where γ is the minimum seed size. The weighted generating function of such segments is (1 + *qz* + … +(*qz*)^γ−1^)*pz*, and that of the tail is 1 + *qz* + … + (*qz*)^γ−1^. This gives a construction plan that can be encoded in a transfer matrix.

Reads consist of only two kinds of objects: the segments terminated by ⇓ (or ⇓-segments for short) and the tails, so the dimension of the transfer matrix is 2 × 2. A ⇓-segment can be followed by another ⇓-segment or by the tail. The tail cannot be followed by anything. The expression of the transfer matrix *M*_0_(*z*) is thus

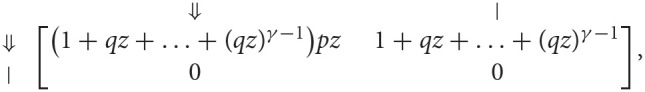



where *p* is the error rate of the sequencer, *q* = 1 − *p* and γ is the minimum seed length. In the representation above, the different types of segments are identified by their terminator, indicated in the margins for clarity.

The entries of *M*_0_(*z*) correspond to sequences of one segment with no seed. Likewise, the entries of $M_{0}{\left(z\right)}^{s}$ correspond to sequences of *s* segments with no seed and the entries of $M_{0}\left(z\right)+M_{0}{\left(z\right)}^{2}+\ldots =M_{0}\left(z\right)\cdot {\left(I-M_{0}\left(z\right)\right)}^{-1}$ correspond to sequences of any number of segments with no seed. The entry of interest is the top right term, associated with terminators ⇓ and |. To see why, observe that every read can be prepended by ⇓-segments and only by those (not by a tail). Thus, reads are precisely the sequences of segments that can follow the symbol ⇓ and that are terminated by a tail, whose weighted generating function is the top right entry of the matrix. In Appendix B.1, we show that this term is equal to

(2) $$\frac{1+qz+\ldots +{\left(qz\right)}^{\gamma -1}}{1-(1+qz+\ldots +{\left(qz\right)}^{\gamma -1})pz}.$$

This function can be expanded as a Taylor series $a_{0}+a_{1}z+a_{2}z^{2}+\ldots$, but the coefficients *a*_0_, *a*_1_, *a*_2_, … are unknown. By construction, *a*_*k*_ is the quantity of interest, i.e., it is the probability that a read of size *k* does not contain an on-target seed of minimum size γ, so we now need to extract the Taylor coefficients from expression (2). There are several methods to do so; the one we choose here is to build a recurrence equation. In Appendix B.2, we show that

(3) $$a_{k}=\{\begin{array}{ll} 1 & \text{ if }k<\gamma ,\\ 1-q^{\gamma } & \text{ if }k=\gamma ,\\ a_{k-1}-pq^{\gamma }\cdot a_{k-\gamma -1} & \text{ otherwise.} \end{array}$$

Note that for *k* < γ, the read is too short so the probability that it contains no seed is 1; for *k* = γ, the read contains a seed if and only if it has no error, which occurs with probability *q*^γ^.

The terms of interest can be computed recursively using expression (3). This approach is very efficient because every iteration involves at most one multiplication and one subtraction. Also, the default floating-point arithmetic on modern computers gives sufficient precision to not worry about numeric instability for the problems considered here (we rarely need to compute those probabilities for reads above 500 nucleotides).

This example shows how the symbolic approach yields a non-trivial and yet simple algorithm to compute the probability that a read of size *k* does not contain an on-target exact seed.

### 3.5. Example 2: On-Target Spaced Seeds

The goal of our second example is to exhibit the mechanisms of our strategy in a more complex setting. In this example we propose to compute the probability that a read of size *k* contains a match for the spaced seed “11*1*1.” This problem has no concrete application, but it illustrates in a relatively simple way the general methodology to deal with spaced seeds.

Here we proceed exactly as in the previous section, replacing nucleotides by the symbols from the mismatch alphabet $A_{0}=\left\{□,|,⇓\right\}$. Recall that □ represents a correct nucleotide, ⇓ represents an erroneous nucleotide and | is the special terminator that marks the end of the read. As explained in the previous example, the weighted generating functions of the symbols □, ⇓ and | are *qz*, *pz*, and 1, respectively, where *p* is the probability of a sequencing error and *q* = 1 − *p*. We again decompose reads into unique sequences of segments, where a segment is the concatenation of zero or more □ symbols with a terminator ⇓, or | for the last segment also called the tail.

So far everything is identical to the previous example, the difference is how we characterize reads that do not contain a seed. This is where the transfer matrix comes in handy. We define four abstract states that represent how the end of the segment matches the seed model “11*1*1.” The states are represented as ⇓, □□⇓, □□⇓□⇓, and □□□□⇓. All the states finish with ⇓ because they correspond to the end of one or more internal segments, which are always terminated by ⇓. To these states we add | for the tail.

To fill the transfer matrix, we need to indicate how the segments bring the read from a state to another. We illustrate how this is done with the transitions from state ⇓. This state indicates that the longest alignment of the previous segment with the seed model has zero nucleotide. If the next segment is ⇓ or □⇓, the longest alignment with the seed model will again have length 0 and the state will remain ⇓. If the next segment is □□⇓ or □□□⇓, the last three nucleotides align with the beginning of the seed model “11*,” bringing the read to the state □□⇓. If the next segment is □□□□⇓ or □□□□□⇓, the last five nucleotides align with “11*1*,” bringing the read to the state □□□□⇓. Finally, segments with more than five □ symbols are disallowed because they create a match for the seed. In all of these segments, the ⇓ terminator can be replaced by | to indicate the end of the read.

Recall from the previous section that the weighted generating function of a segment with *i* symbols □ and a terminator ⇓ is (*qz*)^*i*^*pz*, and that the weighted generating function of a tail with *i* symbols □ is (*qz*)^*i*^. With this information we can fill the row of the transfer matrix that corresponds to state ⇓. With the same logic, we can also fill the other entries of the transfer matrix, making sure that we exclude all the possible matches with the seed. Doing this term by term, we obtain the transfer matrix *M*_◇_(*z*) equal to

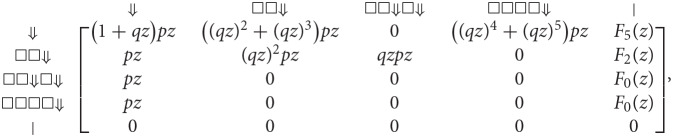



where

$$F_{i}(z)=1+qz+\ldots +{\left(qz\right)}^{i}.$$

The entries of $M_{◇}\left(z\right)+M_{◇}{\left(z\right)}^{2}+\ldots =M_{◇}\left(z\right)\cdot {\left(I-M_{◇}\left(z\right)\right)}^{-1}$ correspond to sequences of any number of segments with no match for the seed. The entry of interest is the top right term, associated with states ⇓ and |. To see why, observe that every read can be prepended by segments finishing in state ⇓ and only by those, otherwise the read would already match some part of the seed before the first nucleotide.

The matrix *M*_◇_(*z*) is simple enough that $M_{◇}\left(z\right)\cdot {\left(I-M_{◇}\left(z\right)\right)}^{-1}$ can be computed explicitly using a computer algebra system. The weighted generating function of interest is found to be the ratio of two polynomials *P*(*z*)/*Q*(*z*) where *P* has degree 9 and *Q* has degree 10. From there, one can proceed as in Appendix B.2 to obtain a recurrence of order 10 that gives the coefficient *a*_*k*_ in the Taylor expansion of *P*(*z*)/*Q*(*z*). By construction, this coefficient *a*_*k*_ is the probability that a read of length *k* does not contain any match for the spaced seed “11*1*1.” Alternatively, one can compute *a*_*k*_ directly from the powers of *M*_◇_(*z*) as explained in the next section.

This approach can be applied for any spaced seed. A seed model with *m* don't-care positions generates 2^*m*^ possible states and the dimension of the associated transfer matrix is 2^*m*^ + 1 (one state is reserved for the tail). For large *m* it is impossible to compute ${\left(I-M_{◇}\left(z\right)\right)}^{-1}$ analytically but one can still compute the powers of *M*_◇_(*z*) efficiently because the matrix is sparse. So in general, the computational method introduced in the next section is more adapted.

### 3.6. Example 3: On-Target Skip Seeds

Let us now go through an example that will be important later. Here we devise a method to compute the probability that a read contains no on-target skip seed. Using the same strategy as in the previous example, we start by recoding the reads using a specialized alphabet to solve this problem.

We need to know whether a nucleotide is a sequencing error, but this time we also need to know its phase in the repeated cycles of skipped positions. For this, we define the skip-*n* alphabet $A_{n}=\left\{□,|,{⇓}_{0},{⇓}_{1},\ldots ,{⇓}_{n}\right\}$. Again, the symbol □ represents a correct nucleotide and the symbol | is a terminator added at the end of the read. The symbols ⇓_*j*_ (0 ≤ *j* ≤ *n*) represent sequencing errors and *j* indicates the number of nucleotides until the next non-skipped position (i.e., *j* = 0 for nucleotides immediately before a non-skipped position and *j* = *n* for nucleotides at a non-skipped position).

**Figure 7:**
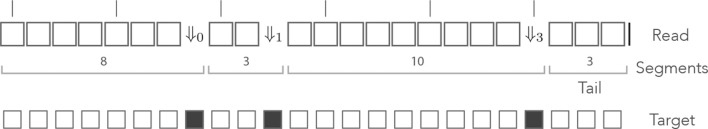
The skip encoding. The read of Figure 6 is represented in the skip-3 alphabet. The symbols ⇓_*j*_(*j* = 1, 2, 3) represent mismatches against the target (they are erroneous nucleotides) and the □ symbol represents a match (it is a correct nucleotide). The vertical bars indicate non-skipped positions (the potential start of a seed). The number *j* in ⇓_*j*_ indicates the number of nucleotides until the next non-skipped position. For *j* = 0 the next nucleotide is not skipped. Other features are as in Figure 6.

As per definition 7, segments in this alphabet are sequences of 0 or more □ symbols followed by any of the symbols ⇓_*j*_ or by the symbol |. Given that this decomposition is unique, we can again view a read as a sequence of segments with a tail. The example of Figure 6 is shown again in Figure 7, where segments in the mismatch alphabet have been replaced by segments in the skip-3 alphabet.

The probability of occurrence of a sequencing error is *p*, so every symbol ⇓_*j*_ has the same weighted generating function *pz*—provided the next non-skipped position is at distance *j*, otherwise the weighted generating function is 0. The weighted generating function of the symbol □ is again *qz*, so a segment with *i* symbols □ followed by the symbol ⇓_*j*_ has weighted generating function (*qz*)^*i*^*pz*—if the next non-skipped position is at distance *j*, otherwise it is 0. As in the previous example, a tail segment with *i* symbols □ followed by the symbol | has weighted generating function (*qz*)^*i*^.

This case is more complex than the previous one: reads without on-target skip seed of minimum size γ can contain segments with γ or more □ symbols. For instance, the read shown in Figure 7 contains a stretch of 9 nucleotides without errors but it has no seed of minimum size γ = 9. More generally, if there is a sequencing error *i* nucleotides before the next non-skipped position, there can be up to γ + *i* − 1 symbols □ in a row.

Expressed in different words, it is possible to append segments with up to γ + *i* − 1 symbols □ after segments terminated by ⇓_*i*_ (⇓_*i*_-segments for short). Each of those γ + *i* possible segments is associated with a different terminator, depending on how far ahead the next non-skipped position lies. In Figure 7, for instance, the second segment is terminated by ⇓_1_ because there is 1 nucleotide before the next non-skipped position. If the segment were 1 nucleotide longer, the terminator would have to be ⇓_0_.

The main issue is that segments of different lengths can be terminated by the same symbol. Going back to Figure 7, the third segment has length 10 and is terminated by ⇓_3_. It would also be terminated by ⇓_3_ if it had length 2 or length 6. In the general case, the entries of the transfer matrix show some periodicity modulo *n* + 1.

Denote *H*_*i, j*_(*z*) the weighted generating function of ⇓_*j*_-segments that can follow a ⇓_*i*_-segment. The total number of segments that can follow a ⇓_*i*_-segment is γ + *i*. Among them, the shortest ⇓_*j*_-segment has size ℓ_0_ to be determined below, and the others have sizes ℓ_0_ + (*n* + 1), ℓ_0_ + 2(*n* + 1), …, ℓ_0_ + *m*(*n* + 1), for some integer *m*. This *m* is the largest number such that ℓ_0_ + *m*(*n* + 1) ≤ γ + *i*, so *m* = ⌊(γ + *i* − ℓ_0_)/(*n* + 1)⌋, where ⌊ … ⌋ is the “floor” function.

Those segments follow a ⇓_*i*_-segment, so they start *i* nucleotides before the next non-skipped position. The shortest following segment that ends *j* nucleotides before a non-skipped position has length ℓ_0_ = *i* − *j* if *i* > *j*, and *n* + 1 − *j* + *i* otherwise. This is equivalent to defining ℓ_0_ as *x* + 1 where *x* is the number of □ symbols of the shortest ⇓_*j*_-segment, i.e., *x* = *i* − *j* − 1 (mod *n* + 1).

With these notations, the shortest ⇓_*j*_-segment consists of *x* symbols □ followed by the ⇓_*j*_ terminator, so it has weighted generating function (*qz*)^*x*^*pz*. The other segments have a multiple of *n* + 1 extra □ symbols so their weighted generating functions are (*qz*)^*x*+(*n*+1)^*pz*, (*qz*)^*x*+2(*n*+1)^*pz*, …, (*qz*)^*x*+*m*(*n*+1)^*pz*. Summing over those cases, we finally obtain

(4) $$\begin{array}{l} H_{i,j}(z)={\left(qz\right)}^{x}(1+{\left(qz\right)}^{n+1}+\ldots +{\left(qz\right)}^{m(n+1)})pz,\\ \text{ where }x=i-j-1\left(\mathrm{mod}n+1\right),\text{ and}\\ \;m=\left\lfloor \frac{\gamma +i-1-x}{n+1}\right\rfloor . \end{array}$$

Denote *J*_*i*_(*z*) the weighted generating function of tail segments that can follow a ⇓_*i*_-segment. There are γ + *i* such tails, each consisting of 0 to γ + *i* − 1 symbols □ followed by the special | terminator for the end of the read. The | symbol has size 0 so its weighted generating function is 1. Once again summing over the different cases, we obtain

(5) $$J_{i}(z)=1+qz+{\left(qz\right)}^{2}+\ldots +{\left(qz\right)}^{\gamma +i-1}.$$

Finally, the expression of *M*_*n*_(*z*) the transfer matrix of reads in the skip-*n* alphabet is

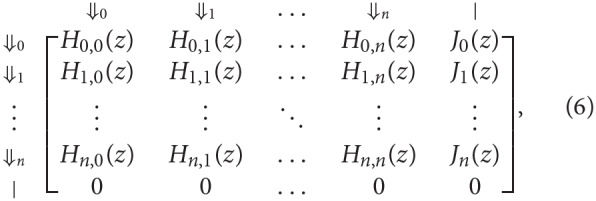



where *p* is the error rate of the sequencer, *q* = 1 − *p*, *n* is the number of skipped nucleotides between potential seeds, γ is the minimum seed length, and polynomials *H*_*i, j*_(*z*) and *J*_*i*_(*z*) are defined in expressions (4) and (5).

In Appendix B.3, we present a transfer matrix with a simpler expression that will prove useful in section 5. The version of Appendix B.3 is simpler, but the expression above has some advantages that will be explained below.

The weighted generating function of interest is the top right entry of the matrix $M_{n}\left(z\right)+M_{n}{\left(z\right)}^{2}+\ldots =M_{n}\left(z\right)\cdot {\left(I-M_{n}\left(z\right)\right)}^{-1}$. To see why, observe that, at the start of every read, the next nucleotide is a non-skipped position, so every read can be prepended by ⇓_0_-segments and only by those. Thus, reads are precisely the sequences of segments that can follow the symbol ⇓_0_ and that are terminated by a tail, whose weighted generating function is the entry of the matrix associated with terminators ⇓_0_ and |.

By construction, the Taylor expansion of the top right term in the matrix $M_{n}\left(z\right)\cdot {\left(I-M_{n}\left(z\right)\right)}^{-1}$ contains the probabilities of interest. More specifically, if the Taylor series of this term is $a_{0}+a_{1}z+a_{2}z^{2}+\ldots$, then *a*_*k*_ is the probability that a read of size *k* contains no skip-*n* seed of minimum size γ.

But *M*_*n*_(*z*) is too complex to find a closed expression for $M_{n}\left(z\right)\cdot {\left(I-M_{n}\left(z\right)\right)}^{-1}$ or its top right term. Instead, we return to the definition $M_{n}\left(z\right)+M_{n}{\left(z\right)}^{2}+\ldots$ and show in Appendix B.4 that the coefficient of interest, *a*_*k*_, only depends on the first *k* + 1 terms of the sum. So for reads of size *k* or lower, we only need to compute the matrix $M_{n}\left(z\right)+M_{n}{\left(z\right)}^{2}+\ldots +M_{n}{\left(z\right)}^{k+1}$ and work out the Taylor expansion of the top right term.

But we can do better: since we are only interested in the coefficients up to order *k*, we can perform all algebraic operations on truncated polynomials of order *k*, i.e., we discard the coefficients of order *k* + 1 or greater when multiplying two polynomials.

But we can do even better: a read with *s* + 1 segments contains *s* errors, so the top right entry of $M_{n}{\left(z\right)}^{s+1}$ is the weighted generating function of reads with *s* errors that have no seed of minimum size γ. Computing the partial sum $M_{n}\left(z\right)+M_{n}{\left(z\right)}^{2}+\ldots +M_{n}{\left(z\right)}^{s}$ instead of $M_{n}\left(z\right)+M_{n}{\left(z\right)}^{2}+\ldots +M_{n}{\left(z\right)}^{k+1}$ corresponds to neglecting reads with *s* or more errors. For *s* sufficiently large, such reads are exceedingly rare so we can obtain accurate estimates without computing all the powers of *M*_*n*_(*z*) up to order *k*.

The number of errors *X* in a read of size *k* has a Binomial distribution *X* ~ *B*(*k, p*). From Arratia and Gordon (1989) we can bound the probabilities of the tail with the expression

(7) $$Pr(X\geq s)\leq \exp\left((s-k)\log\frac{k-s}{k(1-p)}-s\log\frac{s}{kp}\right).$$

Using the formula above, we can thus bound the probability that a read has *s* + 1 or more segments. We compute $M_{n}\left(z\right)+M_{n}{\left(z\right)}^{2}+\ldots +M_{n}{\left(z\right)}^{s+1}$ where the weighted generating functions have been replaced by truncated polynomials and we extract the top right entry. When the right-hand side of expression (7) is lower than a set fraction ε of the current value of *a*_*k*_, we stop the computations. Typically ε = 0.01 so this method ensures that the probabilities that a read of size *k* has no on-target skip seed are accurate to within 1%.

With $M_{n}^{*}\left(z\right)$, the transfer matrix of Appendix B.3, the top right entry of $M_{n}^{*}{\left(z\right)}^{s+1}$ is not the weighted generating function of reads with *s* errors, so (7) is not an upper bound for the neglected terms of the sum. As a consequence, one would have to compute more terms in the partial sum $M_{n}^{*}\left(z\right)+M_{n}^{*}{\left(z\right)}^{2}+\ldots +M_{n}^{*}{\left(z\right)}^{s+1}$ to reach the same accuracy. The transfer matrix shown in expression (6) is not the simplest, but it has the benefit of requiring fewer iterations.

***Remark 1***. *Observe that when n* = 0 *the matrix M*_*n*_(*z*) *is identical to the matrix M*_0_(*z*) *of section 3.4. This is consistent with the fact that exact seeds are skip-0 seeds*.

## 4. Off-Target Exact Seeds

We now turn our attention to the problem of computing the probability that the seeding process is off-target when using exact seeds—recall from section 1.3 that off-target seeding means that the candidate set contains a duplicate but not the target.

If there is no duplicate (i.e., *N* = 0), seeding cannot be off-target, it can only be on-target or null. So from here we assume that the target has *N* ≥ 1 duplicates. Let *S*_0_ denote the event that there is an on-target seed and let *S*_*j*_ denote the event that there is a seed for the *j*-th duplicate. We are thus interested in computing $P\left({\bar{S}}_{0}\cap \left(S_{1}\cup \ldots \cup S_{N}\right)\right)$, where ${\bar{S}}_{j}$ denotes the complement of the event *S*_*j*_. First observe that

(8) $$P({\bar{S}}_{0}\cap (S_{1}\cup \ldots \cup S_{N}))=P({\bar{S}}_{0})-P({\bar{S}}_{0}\cap {\bar{S}}_{1}\cap \ldots \cap {\bar{S}}_{N}).$$

Since the duplicates are assumed to evolve independently of each other and through the same mutagenesis process, the events *S*_*j*_ (1 ≤ *j* ≤ *N*) are independent and identically distributed conditionally on ${\bar{S}}_{0}$. We can thus write

(9) $$\begin{array}{l} P({\bar{S}}_{0}\cap \ldots \cap {\bar{S}}_{N})=P({\bar{S}}_{0})\cdot P({\bar{S}}_{1}\cap \ldots \cap {\bar{S}}_{N}|{\bar{S}}_{0})\\ \;=P({\bar{S}}_{0})\cdot P{\left({\bar{S}}_{1}|{\bar{S}}_{0}\right)}^{N}=P({\bar{S}}_{0})\cdot {\left(\frac{P({\bar{S}}_{0}\cap {\bar{S}}_{1})}{P({\bar{S}}_{0})}\right)}^{N}. \end{array}$$

Combining the two equations above, we obtain

(10) $$P({\bar{S}}_{0}\cap (S_{1}\cup \ldots \cup S_{N}))=P({\bar{S}}_{0})-P({\bar{S}}_{0})\cdot {\left(\frac{P({\bar{S}}_{0}\cap {\bar{S}}_{1})}{P({\bar{S}}_{0})}\right)}^{N}.$$

Hence, the probability that seeding is off-target is a function of just two quantities: $P\left({\bar{S}}_{0}\right)$ and $P\left({\bar{S}}_{0}\cap {\bar{S}}_{1}\right)$. The first is the probability that the read has no seed for the target, which we have already computed in section 3.4 using recursive expression (3). We now need to find a way to compute $P\left({\bar{S}}_{0}\cap {\bar{S}}_{1}\right)$.

***Remark 2***. *Observe that expression (9) is the probability of null seeding (the read contains no seed for the target or any of its duplicates). Since it is also a function of just* $P\left({\bar{S}}_{0}\right)$ *and* $P\left({\bar{S}}_{0}\cap {\bar{S}}_{1}\right)$, *it can be computed at no additional cost. This probability is less relevant than the probabilities that the seeding process is on-target or off-target, but at times, it may be useful to know the probability that a read is not mappable, especially when reads are relatively short*.

### 4.1. The Dual Encoding

$P\left({\bar{S}}_{0}\cap {\bar{S}}_{1}\right)$ is the probability that the read has no seed for the target or for the first duplicate—numbering is arbitrary here, the first duplicate can be any fixed duplicate. As in section 3, we first recode the reads using a specialized alphabet to simplify the problem.

It will be useful to consider a more general problem where we have two sequences of interest labeled + and −. The + sequence stands for the target and that the − sequence stands for its duplicate. We then define the dual alphabet ${\tilde{A}}_{0}=\left\{□,|,{↓}_{/1}^{-}\;,{↓}_{/2}^{-}\;,\ldots ,{↓}_{/1}^{+},{↓}_{/2}^{+},\ldots ,⇓\right\}$. The symbols ${↓}_{/j}^{-}$ (*j* ≥ 1) signify that the nucleotide is a mismatch against the − sequence only, the symbols ${↓}_{/j}^{+}$ (*j* ≥ 1) signify that it is a mismatch against the + sequence only, and the symbol ⇓ signifies that it is a mismatch against both. As before, every other nucleotide is replaced by the symbol □, and the terminator | is appended to the end of the read. We again define reads as sequences of segments (zero or more □ symbols followed by a terminator), except that now the terminators are the symbols ${↓}_{/j}^{-}$, ${↓}_{/j}^{+}$ (*j* ≥ 1) and ⇓. The tail, as usual, is terminated by the symbol |.

The index *j* in the symbol ${↓}_{/j}^{-}$ indicates the match length of the + sequence (note that this is not the same as the number of □ symbols in the segment). Likewise, the index *j* in the symbol ${↓}_{/j}^{+}$ indicates the match length of the − sequence. For instance, the symbol ${↓}_{/7}^{-}$ indicates that the nucleotide is a mismatch against the − sequence, that it is a match for the + sequence, and that the six preceding nucleotides were also a match for the + sequence (but the nucleotide before that was a mismatch against the + sequence). The terminators thus encode the local state of the read.

**Figure 8:**
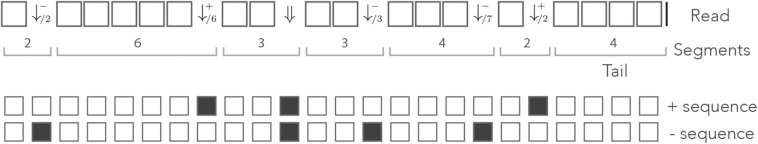
Example of dual encoding. An example of read is represented in the dual alphabet. The symbols ${↓}_{/j}^{-}$ (*j* ≥ 1) represent a mismatch against the − sequence, the symbols ${↓}_{/j}^{+}$ (*j* ≥ 1) represent a mismatch against the + sequence, and the symbol ⇓ represents a mismatch against both. The index *i* is the match length of the sequence that is not mismatched. The symbolic + and − sequences are represented below, where an open square stands for a match and a closed square stands for a mismatch.

Figure 8 shows an example of read in the dual encoding. The + and − sequences are shown below the read, with matches represented as open squares and mismatches as closed squares. It is visible from this example that symbols ${↓}_{/j}^{-}$ and ${↓}_{/j}^{+}$ alternate whenever the mismatches hit different sequences. The symbol ⇓ occurs only when a nucleotide is a double mismatch.

Let us assume that for each nucleotide, *a* is the probability that the read matches both sequences, *b* is the probability that it matches only the + sequence, *c* is the probability that it matches only the − sequence and *d* is the probability that it matches neither. Since there are no other cases, we have *a* + *b* + *c* + *d* = 1.

With these definitions, the weighted generating functions of the symbols □, ${↓}_{/j}^{-}$, ${↓}_{/j}^{+}$ (*j* ≥ 1) and ⇓ are *az*, *bz*, *cz*, and *dz*, respectively. The next sections clarify how this is used to compute the weighted generating functions of interest.

### 4.2. Segments Following ⇓

After a ⇓ terminator, the match counter for both sequences is reset; the following segment can thus have up to γ − 1 matches for any of the two sequences. Each match corresponds to the □ symbol with weighted generating function *az*. The terminators ⇓ and | have respective generating function *dz* and 1 (recall that the tail symbol has size 0), so if the next terminator is ⇓ or |, the segments have weighted generating functions (1 + *az* + … +(*az*)^γ−1^)*dz* or 1 + *az* + … +(*az*)^γ−1^, respectively.

If the next terminator is ${↓}_{/j}^{-}$, there is a match of length *j* for the + sequence, so the segment contains *j* − 1 symbols □ plus the terminator (which also matches the + sequence). The weighted generating function is thus (*az*)^*j*−1^*bz*. By the same rationale, if the next terminator is ${↓}_{/j}^{+}$, the weighted generating function of the segment is (*az*)^*j*−1^*cz*.

The terminators ${↓}_{/j}^{+}$ and ${↓}_{/j}^{-}$ are disallowed for *j* ≥ γ because this would create a seed for at least one of the sequences.

### 4.3. Segments Following ${↓}_{/i}^{+}$

At a ${↓}_{/i}^{+}$ terminator, the match length of the + sequence is 0 and the match length of the − sequence is *i*. The next segment can thus have up to γ − 1 matches for the + sequence, but only γ − *i* − 1 matches for the − sequence, lowering the maximum size of the segment. If the next terminator is ⇓ or |, the segments have weighted generating functions (1 + *az* + … +(*az*)^γ−*j*−1^)*dz* and 1 + *az* + … +(*az*)^γ−*j*−1^, respectively.

If the next terminator is ${↓}_{/j}^{-}$, the weighted generating function is (*az*)^*j*−1^*bz* as in the previous section. The difference is that the terminators ${↓}_{/j}^{-}$ are allowed only for 1 ≤ *j* ≤ γ − *i*, otherwise this would create a seed for the − sequence.

If the next terminator is ${↓}_{/j}^{+}$, the situation is slightly more complex because this imposes *i* < *j* ≤ γ − 1. Indeed, there were already *i* matches for the − sequence at the terminator ${↓}_{/i}^{+}$, and there will be more at the end of the following segment because it has no mismatch for the − sequence. Taking this into account, we see that the weighted generating function of those segments is (*az*)^*j*−*i*−1^*cz* with *i* < *j* ≤ γ − 1.

### 4.4. Segments Following ${↓}_{/i}^{-}$

We can find the weighted generating functions by just reversing the + and − signs in the previous section. This way, we see that the weighted generating function of the segments terminated by ⇓ or | are (1 + *az* + … +(*az*)^γ−*i*−1^)*dz* and 1 + *az* + … +(*az*)^γ−*i*−1^, respectively.

Likewise, the weighted generating function of the segments terminated by ${↓}_{/j}^{+}$ is (*az*)^*j*−1^*cz*, where 1 ≤ *j* ≤ γ − *i*; and the weighted generating function of the segments terminated by ${↓}_{/j}^{-}$ is (*az*)^*j*−*i*−1^*bz* where *i* < *j* ≤ γ − 1.

### 4.5. Transfer Matrix

We now have all the elements to specify the transfer matrix of reads with no seed for either sequence. Recall that *a* is the probability of a double match, *b* is the probability of a mismatch only against the − sequence, *c* is the probability of a mismatch only against the + sequence and *d* is the probability of a double mismatch. For notational convenience, we define

(11) $$\begin{array}{l} \;r_{i}^{+}(z)={\left(az\right)}^{i}cz,\\ \;r_{i}^{-}(z)={\left(az\right)}^{i}bz,\\ R_{i}(z)=(1+az+\ldots +{\left(az\right)}^{i})dz,\\ \;F_{i}(z)=1+az+\ldots +{\left(az\right)}^{i}. \end{array}$$

With these notations, the information from the previous sections can be summarized in the transfer matrix ${\tilde{M}}_{0}\left(z\right)$ equal to

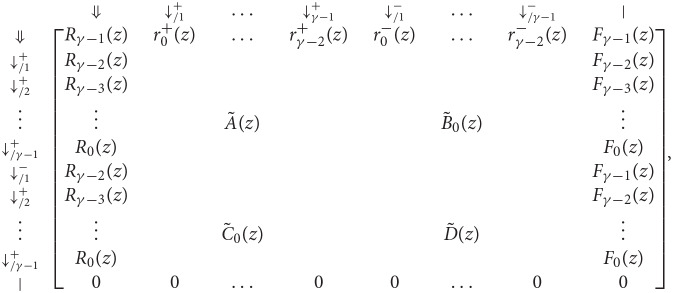



where γ is the minimum seed length, and where Ã(*z*), ${\tilde{B}}_{0}\left(z\right)$, ${\tilde{C}}_{0}\left(z\right)$, and $\tilde{D}\left(z\right)$ are matrices of dimensions (γ − 1) × (γ − 1) that are defined as

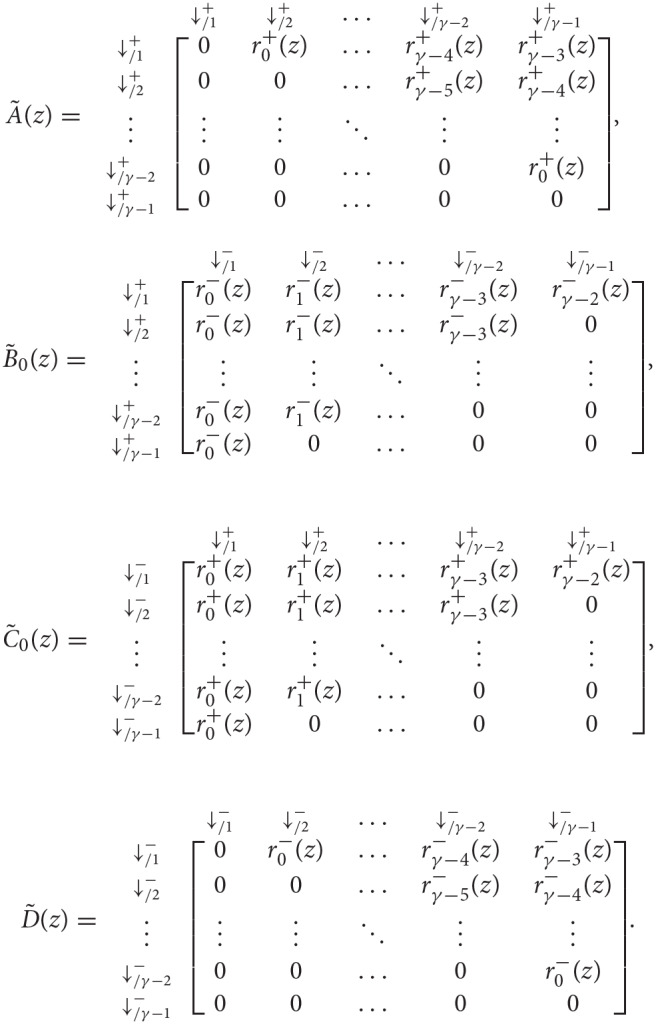



As before, the term of interest is the top right entry of ${\tilde{M}}_{0}\left(z\right)\cdot {\left(I-{\tilde{M}}_{0}\left(z\right)\right)}^{-1}$. To see why, observe that every read can be prepended by ⇓-segments and only by those (every other terminator would imply that one of the two sequences has a nonzero match size at the start of the read). Thus, reads are precisely the sequences of segments that can follow the symbol ⇓ and that are terminated by a tail, the weighted generating function of which is the top right entry of the matrix.

${\tilde{M}}_{0}\left(z\right)$ is too complex to compute a closed expression of ${\tilde{M}}_{0}\left(z\right)\cdot {\left(I-{\tilde{M}}_{0}\left(z\right)\right)}^{-1}$. It is easier to proceed as in section 3.6 and to compute the powers of ${\tilde{M}}_{0}\left(z\right)$ up to a finite value. This is done once again using the arithmetic of truncated polynomials. Since each segment except the tail contains a mismatch against at least one sequence, the top right entry of ${\tilde{M}}_{0}{\left(z\right)}^{s+1}$ is the weighted generating function of reads that contain *s* mismatches (where double mismatches count as one). We thus define $\tilde{p}$ as the upper bound on the probability of a mismatch, *i.e*. $\tilde{p}=\max\left\{b,c,d\right\}$. The updated formula (7) now gives an upper bound of the probability that a read of size *k* contains *s* or more mismatches as

$$Pr(X\geq s)\leq \exp\left((s-k)\log\frac{k-s}{k(1-\tilde{p})}-s\log\frac{s}{k\tilde{p}}\right).$$

With this upper bound, we can compute the terms of the matrix partial sums ${\tilde{M}}_{0}\left(z\right)+{\tilde{M}}_{0}{\left(z\right)}^{2}+\ldots +{\tilde{M}}_{0}{\left(z\right)}^{s}$ until the ignored terms become negligible, *i.e*. until we can be sure that the coefficient of interest *a*_*k*_ is accurate to within chosen ε.

Now returning to the problem of computing $P\left({\bar{S}}_{0}\cap {\bar{S}}_{1}\right)$, the + sequence is interpreted as the target and the − sequence as the duplicate. Based on the assumptions of the error model presented in section 3.1, this implies that *a* = (1 − *p*)(1 − μ), *b* = (1 − *p*)μ, *c* = *pμ*/3, and *d* = *p*(1 − μ/3).

With these values, we can fully specify the matrix ${\tilde{M}}_{0}\left(z\right)$, and compute its powers until the estimate of the coefficient of interest *a*_*k*_ is accurate enough, finally giving a numerical value for $P\left({\bar{S}}_{0}\cap {\bar{S}}_{1}\right)$.

### 4.6. Illustration

We illustrate the strategy delineated above for reads of size *k* = 50 sequenced with an instrument with error rate *p* = 0.01, when using exact seeds of size γ = 19.

**Figure 9:**
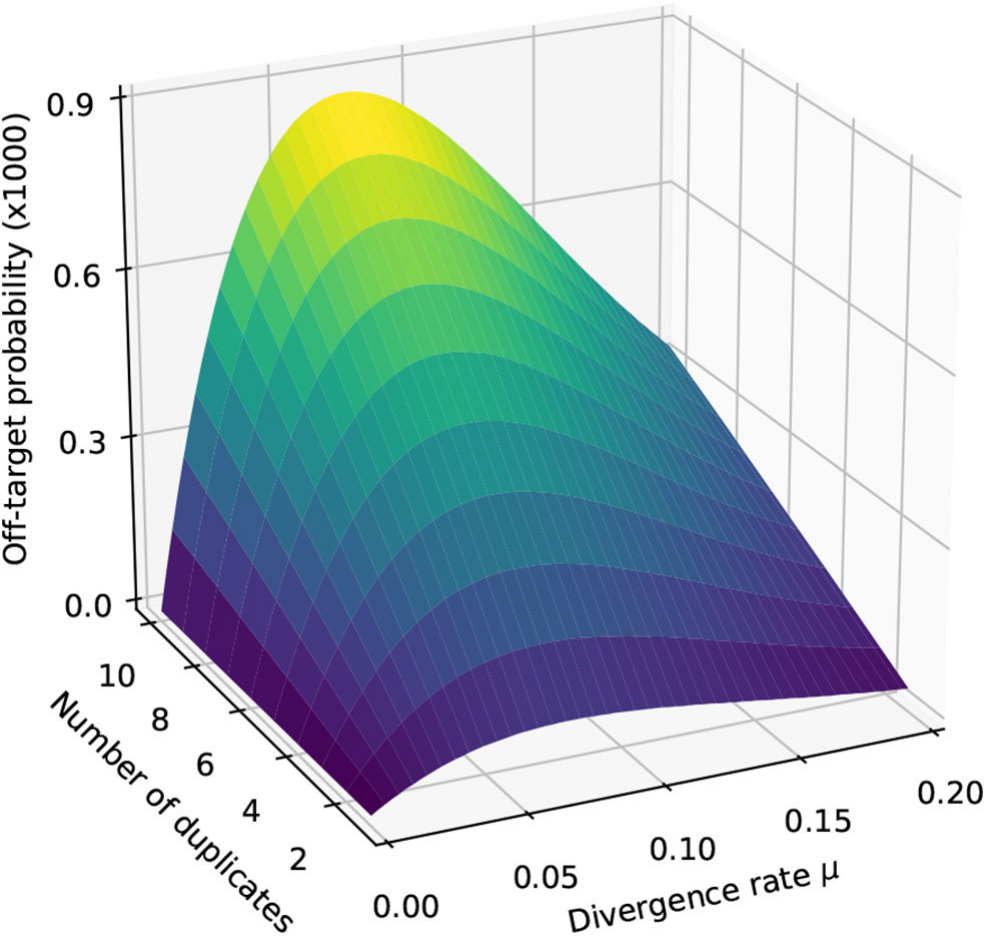
Off-target seeding probabilities (exact seeds). The surfaces show the probability that seeding is off-target as a function of the divergence rate μ and of the number *N* of duplicates. The results are shown for exact seeds of size γ = 19 and for reads of size *k* = 50 nucleotides sequenced with an error rate *p* = 0.01. The divergence rate μ is defined as the probability that a given duplicate differs from the target at any given position. Note the factor 1,000 in the scale of the z-axis.

Figure 9 shows the result for a number of duplicates *N* from 1 to 10 and for a divergence rate μ from 0 to 0.20. The first observation is that the probability that seeding is off-target increases with *N*. This is also clear from expression (10). This can also be understood intuitively because the probability of not seeding the target is fixed and the probability of having an empty candidate set decreases as *N* increases. As a result the probability that the candidate set contains only invalid candidates increases with *N*.

The second observation is that there exists a “worst” value of μ situated around 0.070. When μ is much smaller, the duplicates tend to be exactly identical to the target, making it impossible that there is a seed for a duplicate but no seed for the target. When μ is much larger, the duplicates are far from the target and they are unlikely to be in the candidate set at all. In expression (10), the only term that depends on μ is $P\left({\bar{S}}_{0}\cap {\bar{S}}_{1}\right)$, and it is clear that the minimum of expression (10) corresponds to the maximum of $P\left({\bar{S}}_{0}\cap {\bar{S}}_{1}\right)$. This is why the worst value of μ is the same for every *N*.

## 5. Off-Target Skip Seeds

To compute the probability that the seeding process is off-target when using skip seeds, we observe that the logic of section 4 can be transposed with few modifications. In particular, the probability can be computed through expression (10), where *S*_0_ is the event that the read has a *skip* seed for the target (instead of an exact seed) and *S*_1_ is the event that the read has a *skip* seed for the first duplicate (instead of an exact seed).

We have already seen how to compute $P\left({\bar{S}}_{0}\right)$ in section 3.6, we now need to find a way to compute $P\left({\bar{S}}_{0}\cap {\bar{S}}_{1}\right)$ when using skip seeds.

### 5.1. The Skip Dual Encoding

As before, we recode the reads in a specialized alphabet. We define the skip-*n* dual alphabet as ${\tilde{A}}_{n}=\left\{□,*,|,{⇓}_{0},{⇓}_{1},{⇓}_{2},\ldots ,{⇓}_{n},{↓}_{/1}^{-},{↓}_{/2}^{-},\ldots ,{↓}_{/1}^{+},{↓}_{/2}^{+},\ldots \right\}$. The symbols □, |, ${↓}_{/j}^{-}$ and ${↓}_{/j}^{+}$ (*j* ≥ 1) have the same meaning as in the dual alphabet of section 4.1, i.e., the □ symbol stands for a double match, the | terminator marks the end of the read, the ${↓}_{/j}^{-}$ symbol indicates a mismatch against the − sequence with a match length of size *j* for the + sequence, and conversely the ${↓}_{/j}^{+}$ symbol indicates a mismatch against the + sequence with a match of length *j* for the − sequence. The symbols ⇓_*j*_ (0 ≤ *j* ≤ *n*) indicate that both sequences have match length 0 and that the next non-skipped position is *j* nucleotides further. The symbol * indicates that it does not matter whether the nucleotide is a match or a mismatch, as we will explain below.

**Figure 10:**
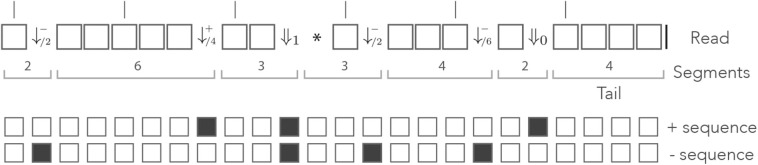
Example of skip dual encoding. The read of Figure 8 is represented in the skip dual alphabet. The vertical bars above the read indicate non-skipped positions. The symbols ${↓}_{/j}^{-}$ and ${↓}_{/j}^{+}$ (*j* ≥ 1) have the same meaning as in the dual alphabet. The symbols ⇓_*j*_ indicate that both sequences have match length 0 and that the next non-skipped position is located *j* nucleotides downstream. Other features are as in Figure 8.

Figure 10 shows the read from Figure 8 represented in the skip-3 dual encoding. It is important to note several differences with Figure 8. The first is that the symbols ⇓_*j*_ (0 ≤ *j* ≤ *n*) are not always associated with double mismatches. For instance, the symbol ⇓_0_ on the right side of the read corresponds to a mismatch for the + sequence only. This happens whenever the + and the − sequences are mismatched in the same interval between non-skipped positions (the mismatches do not need to be on the same nucleotide).

Also observe that the symbol ⇓_1_ is followed by a * symbol, indicating that it does not matter whether the nucleotide is a match or a mismatch for any of the two sequences. After the ⇓_1_ symbol, both sequences have match length 0 and we have to “wait” for a potential new seed one nucleotide further downstream. As in Appendix B.3, the ⇓_*j*_ symbols are followed by *j* symbols *, unless the end of the read comes before the next non-skipped position. After a sequence of * symbols, the read is either finished or at a non-skipped position. In the second case, the is in the same state as after a ⇓_0_ symbol, so we can consider that the * symbol allows the terminators ⇓_*j*_ (1 ≤ *j* ≤ *n*) to just “fast forward” to either ⇓_0_ or |, as shown in Appendix B.3.

As in the previous section, let *a*, *b*, *c*, and *d* denote the probabilities of a double match, a mismatch against −, a mismatch against + and a double mismatch, respectively. With these definitions, the weighted generating functions of the symbols □, ${↓}_{/j}^{-}$ and ${↓}_{/j}^{+}$ (*j* ≥ 1) are *az*, *bz*, and *cz*, respectively. The weighted generating function of the * symbol is *z*, and that of the symbols ⇓_*j*_ (0 ≤ *j* ≤ *n*) will be worked out in the sections below.

### 5.2. Segments Following ⇓_*i*_ (1 ≤ *i* ≤ *n*)

A ⇓_*i*_ terminator is followed by up to *i* symbols *. If there are fewer than *i* symbols *, the read is finished so the segment must be a tail. Recall that the weighted generating function of the | terminator is 1, so the weighted generating function of tail segments is 1 + *z* + … +*z*^*i*−1^.

If there are *i* symbols *, the sequence ends at a non-skipped position, *i.e*. in the same state as after a ⇓_0_ terminator. This is not a segment proper, because there is no terminator, but in the transfer matrix the symbols ⇓_*i*_ (1 ≤ *i* ≤ *n*) project directly to the symbol ⇓_0_ with weighted generating function *z*^*i*^.

### 5.3. Segments Following ⇓_0_

After a ⇓_0_ symbol, all the counters are reset as in the beginning of the read. If the next terminator is |, the segment is a tail and we only have to make sure that it contains fewer than γ symbols □, otherwise this would create a seed. The weighted generating function of tail segments following ⇓_0_ is thus 1 + *qz* + … +(*qz*)^γ−1^.

If the next terminator is a ${↓}_{/j}^{-}$ symbol (*j* ≥ 1), the segment is a match of size *j* for the + sequence. This imposes *j* < γ otherwise the segment would create a seed. Such segments consist of *j* − 1 symbols □ followed by the terminator, so their weighted generating function is (*az*)^*j*−1^*bz* with 1 ≤ *j* < γ.

Conversely, if the next terminator is a ${↓}_{/j}^{+}$ symbol (*j* ≥ 1), we can apply the same rationale to see that the weighted generating function is (*az*)^*j*−1^*cz*, where 1 ≤ *j* < γ.

Finally, if the next terminator is a ⇓_*j*_ symbol (0 ≤ *j* ≤ *n*), we must not only make sure that the segment contains fewer than γ symbols □, but also keep track of the position of the next non-skipped position (stored in index *j*). Here we can follow verbatim the rationale of section 3.6 where we replace the probability of a match (previously *q*) by that of a double match (now *a*), and the probability of a mismatch (previously *p*) by that a double mismatch (now *d*). Replacing the symbols in expression (4), we see that the weighted generating function is (*az*)^*x*^ · (1 + (*az*)^*n*+1^ + … + (*az*)^(*n*+1)*m*^)*dz*, where *x* = *n* − *j* (mod *n* + 1) and *m* = ⌊(γ − 1 − *x*)/(*n* + 1)⌋.

### 5.4. Segments Following ${↓}_{/i}^{-}$

At a ${↓}_{/i}^{-}$ terminator (*i* ≥ 1), the read contains a match of length *i* for the + sequence. If the next segment is a tail, we must make sure that the match length for the + sequence does not exceed γ − 1. This means that we can have up to γ − *i* − 1 symbols □ and thus that the weighted generating function of the tail segments is 1 + (*qz*) + … +(*qz*)^γ−*i*−1^.

If the next terminator is a ${↓}_{/j}^{-}$ symbol, we must have *j* > *i* because there is no mismatch against the + sequence. In this case, we must only make sure that the total match length for the + sequence remains lower than γ. Such segments contain *j* − *i* − 1 symbols □ followed by the terminator so their weighted generating function is (*qz*)^*i*−*j*−1^*bz* with *i* < *j* ≤ γ − 1.

For the last two types of terminators, we must pay attention to the fact that in general, a mismatch against + that follows a mismatch against − can be represented by either a ⇓_*j*_ symbol (0 ≤ *j* ≤ *n*) or a ${↓}_{/j}^{+}$ symbol (*j* ≥ 1). The terminator is ⇓_*j*_ if the two mismatches are within the same interval between non-skipped positions because both sequences locally have match length 0. If the mismatch against + is in another interval, the terminator is ${↓}_{/j}^{+}$ because in that case the − sequence has a positive match length. For now, bear in mind that a mismatch against the + sequence only can produce a ⇓_*j*_ terminator (0 ≤ *j* ≤ *n*), this will be important later.

If the next terminator is ${↓}_{/j}^{+}$ (*j* ≥ 1), then it must be separated from the preceding ${↓}_{/i}^{-}$ terminator by a non-skipped position, which imposes a lower bound on the size of the segment. Since at the ${↓}_{/i}^{-}$ terminator the match length for + was *i*, there must be a non-skipped position *i* nucleotides before. The number of nucleotides from the ${↓}_{/i}^{-}$ terminator to the next non-skipped position is thus *y* = −*i* (mod *n* + 1), so the minimum segment length is *y* + 1. There is also an upper bound on the size of the segment because the match length for the + sequence cannot become higher than γ−1, imposing the length to be lower than γ − *i*. The shortest segment is terminated by ${↓}_{/1}^{+}$, and the longest by ${↓}_{/\gamma -y-i}^{+}$. Finally, the weighted generating function of segments terminated by ${↓}_{/j}^{+}$ following the ${↓}_{/i}^{-}$ terminator is (*az*)^*y*+*j*−1^*cz* with 1 ≤ *j* ≤ γ − *y* − *i*. Note that for some value of *i* and *y*, no *j* can satisfy the last inequality.

Finally, if the next terminator is ⇓_*j*_ (0 ≤ *j* ≤ *n*) we have to distinguish two cases, depending on whether the segment ends with a double mismatch or with a single mismatch against the + sequence. For the first case we can apply the rationale of section 3.6. Recall that the index *j* indicates the number of nucleotides until the next non-skipped position. Let ℓ_0_ be the length of the shortest possible segment. The lengths of the other segments are of the form ℓ_0_ + *n* + 1, …, ℓ_0_ + *m*(*n* + 1), where the integer *m* must be chosen so that the segment does not create a seed. At the ${↓}_{/i}^{-}$ terminator, there is a match of length *i* for the + sequence so *m* is the largest integer such that *i*+ℓ_0_+*m*(*n*+1) < γ, *i.e*. *m* = ⌊(γ − *i* − ℓ_0_)/(*n* + 1)⌋.

The ${↓}_{/i}^{-}$ terminator is *y* = −*i* (mod *n* + 1) nucleotides before the next non-skipped position so the shortest segment has length ℓ_0_ = *y* − *j* if *y* > *j*, and *n* + 1 − *j* + *y* otherwise. This is equivalent to defining ℓ_0_ as *x* + 1 where *x* is the number of □ symbols in the shortest segment, i.e., *x* = −*i* − *j* − 1 (mod *n* + 1). Summing the weighted generating functions of the individual segments, we find (*az*)^*x*^(1 + (*az*)^*n*+1^+…+(*az*)^*m*(*n*+1)^)*dz*.

The last remaining issue is that a mismatch against the + sequence only can produce a ⇓_*j*_ terminator. This happens when the preceding ${↓}_{/i}^{-}$ terminator is not separated from the mismatch by a non-skipped position (in this case both sequences locally have match length 0). The ${↓}_{/i}^{-}$ terminator is located *y* nucleotides before the next non-skipped position, so if the terminator is from ⇓_0_ to ⇓_*y*−1_ we need to add the term (*az*)^*x*^*bz* to the previous weighted generating function. In conclusion, the weighted generating function of segments terminated by ⇓_*j*_ is ${\left(az\right)}^{x}\left(1+{\left(az\right)}^{n+1}+\ldots +{\left(az\right)}^{m\left(n+1\right)}\right)dz+\delta_{i,j}^{+}\left(z\right)$, where $\delta_{i,j}^{+}\left(z\right)={\left(az\right)}^{x}bz$ if *j* < *y* and 0 otherwise.

### 5.5. Segments Following ${↓}_{/i}^{+}$

We can find the weighted generating functions by just reversing the + and − signs in the previous section. This way we can see that the weighted generating function of tail segments is 1 + (*qz*) + … +(*qz*)^γ−*i*−1^.

Likewise, the weighted generating function of segments terminated by ${↓}_{/j}^{+}$ is (*qz*)^*i*−*j*−1^*bz* with *i* < *j* ≤ γ − 1.

The weighted generating function of segments terminated by ${↓}_{/j}^{-}$ is (*az*)^*y*+*j*−1^*dz* with *y* = −*i* (mod *n* + 1) and 1 ≤ *j* ≤ γ − *y* − *i*.

Finally, the weighted generating function of segments terminated by ⇓_*j*_ is ${\left(az\right)}^{x}\left(1+{\left(az\right)}^{n+1}+\ldots +{\left(az\right)}^{m\left(n+1\right)}\right)dz+\delta_{i,j}^{-}\left(z\right)$, where $\delta_{i,j}^{-}\left(z\right)={\left(az\right)}^{x}cz$ if *j* < *y* and 0 otherwise.

### 5.6. Transfer Matrix

We now have all the elements to specify the transfer matrix of reads with no skip-*n* seed for either sequence. Recall that *n* is the number of skipped nucleotides, γ is the minimum seed length, *a* is the probability of a double match, *b* is the probability of a mismatch against the − sequence only, *c* is the probability of a mismatch against the + sequence only and *d* is the probability of a double mismatch. For notational convenience we define

(12) $$N_{i}(z)=1+z+\ldots +z^{i},$$

(13) $$W_{j}(z)={\left(az\right)}^{x}(1+{\left(az\right)}^{n+1}+\ldots +{\left(az\right)}^{(n+1)m})dz,$$

(14) $$\begin{array}{l} \text{ where }x=-j-1\left(\mathrm{mod}n+1\right),\text{ and }m=\left\lfloor \frac{\gamma -1-x}{n+1}\right\rfloor ,\\ U_{i,j}(z)={\left(az\right)}^{x}(1+{\left(az\right)}^{n+1}+\ldots +{\left(az\right)}^{(n+1)m})dz+\{\begin{array}{l} bz\cdot {\left(az\right)}^{x}\text{ if }j<y,\\ 0\text{ otherwise} \end{array}\\ \text{ where }x=-i-j-1\left(\mathrm{mod}n+1\right),y=-i\left(\mathrm{mod}n+1\right),\\ \text{ and }m=\left\lfloor \frac{\gamma -1-i-x}{n+1}\right\rfloor ,\;(14) \end{array}$$

(15) $$\begin{array}{l} V_{i,j}(z)={\left(az\right)}^{x}(1+{\left(az\right)}^{n+1}+\ldots +{\left(az\right)}^{(n+1)m})dz+\{\begin{array}{l} cz\cdot {\left(az\right)}^{x}\text{ if }j\leq x,\\ 0\text{ otherwise} \end{array}\\ \text{ where }x=-i-j-1\;\left(\mathrm{mod}n+1\right),m=\left\lfloor \frac{\gamma -1-i-x}{n+1}\right\rfloor . \end{array}$$

With these notations, the information from the previous sections can be summarized in the transfer matrix ${\tilde{M}}_{n}\left(z\right)$ equal to

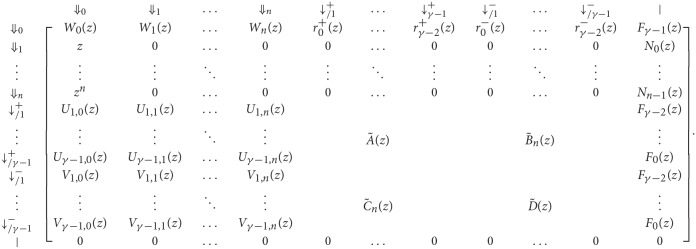



The matrices Ã(*z*) and $\tilde{C}\left(z\right)$ in the expression of ${\tilde{M}}_{n}\left(z\right)$ are the same as in section 4.1. They are reproduced here for convenience.

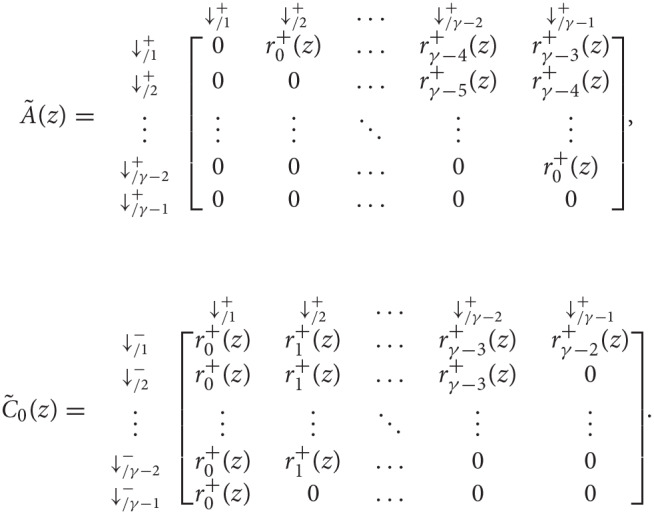



The matrices ${\tilde{B}}_{n}\left(z\right)$ and ${\tilde{C}}_{n}\left(z\right)$ are defined as

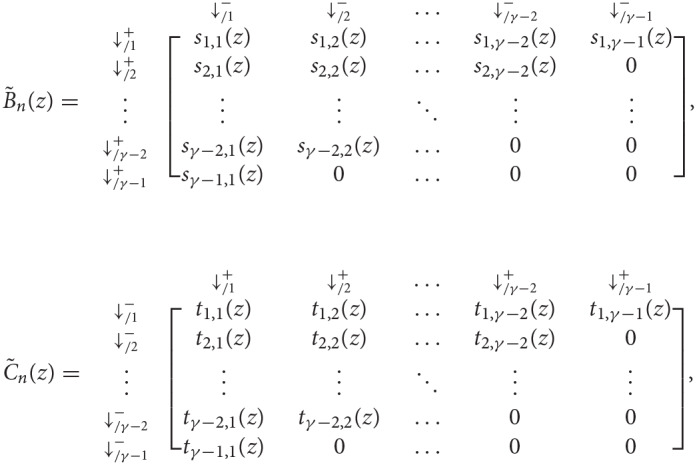



with

$$\begin{array}{l} \;s_{i,j}=\{\begin{array}{ll} cz\cdot {\left(az\right)}^{y+j-1} & \text{ if }i+j+y\leq \gamma \\ 0 & \text{ otherwise,} \end{array}\\ \;t_{i,j}=\{\begin{array}{ll} bz\cdot {\left(az\right)}^{y+j-1} & \text{ if }i+j+y\leq \gamma \\ 0 & \text{ otherwise,} \end{array}\\ \text{where }y=-i\left(\mathrm{mod}n+1\right),\text{ in both cases.} \end{array}$$

Now returning to the problem of computing $P\left({\bar{S}}_{0}\cap {\bar{S}}_{1}\right)$, the + sequence is interpreted as the target and the − sequence as the duplicate. Based on the assumptions of the error model presented in section 3.1, this implies that *a* = (1 − *p*)(1 − μ), *b* = (1 − *p*)μ, *c* = *pμ*/3, and *d* = *p*(1 − μ/3).

The computation is performed as described in section 4.1. We compute the successive powers of ${\tilde{M}}_{n}\left(z\right)$ in the arithmetic of truncated polynomials and stop the iterations using the same criterion. The only modification is that the top right term of ${\tilde{M}}_{n}{\left(z\right)}^{s+1}$ is not the weighted generating function of reads with *s* mismatches (where double mismatches count as one). The reason is that the sequences of * symbols are not terminated by a mismatch. However, there can be at most one sequence of * symbols for each mismatch or double mismatch, so the reads described by the top right term of ${\tilde{M}}_{n}{\left(z\right)}^{s+1}$ have at least ⌊*s*/2⌋ mismatches.

We thus define $\tilde{p}$ as the upper bound on the probability of a mismatch, i.e., $\tilde{p}=\max\left\{b,c,d\right\}$ and use the updated formula (7) from section 4.5

$$Pr(X\geq s)\leq \exp\left((s-k)\text{ log }\frac{k-s}{k(1-\tilde{p})}-s\text{ log }\frac{s}{k\tilde{p}}\right).$$

With this upper bound, we can compute the terms of the partial sums ${\tilde{M}}_{0}\left(z\right)+{\tilde{M}}_{0}{\left(z\right)}^{2}+\ldots +{\tilde{M}}_{0}{\left(z\right)}^{2s+1}$ until the ignored terms become negligible, *i.e*. until we can be sure that the coefficient of interest *a*_*k*_ is accurate to within chosen ε.

***Remark 3***. *Observe that when n = 0 the matrix* ${\tilde{M}}_{n}\left(z\right)$ *is identical to the matrix* ${\tilde{M}}_{0}\left(z\right)$ *of section 4.1, again consistent with the fact that exact seeds are skip-0 seeds. The same applies to* ${\tilde{B}}_{n}\left(z\right)$ *and* ${\tilde{C}}_{n}\left(z\right)$.

### 5.7. Illustration

We illustrate the strategy delineated above using the same settings as in section 4.6 (reads of length *k* = 50, probability of sequencing error *p* = 0.01 and seeds of minimum size γ = 19), except that we replace exact seeds by skip-5 and skip-9 seeds.

**Figure 11:**
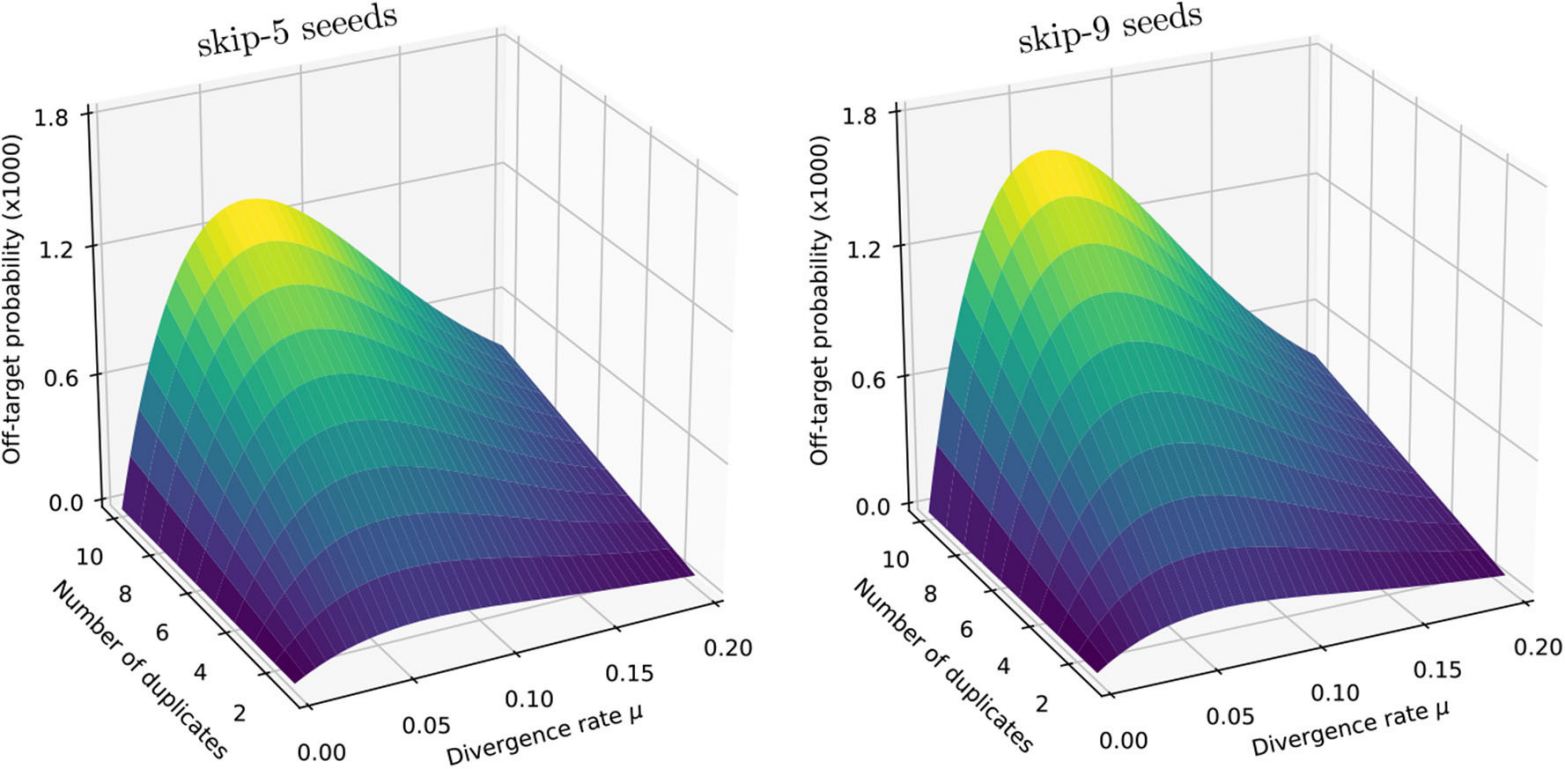
Off-target seeding probabilities (skip seeds). The surfaces show the probability that seeding is off-target for skip-5 and skip-9 seeds of size γ = 19. Read size and error rate are the same as in Figure 9, i.e., *k* = 50 and *p* = 0.01. The divergence rate μ is defined as the probability that a given duplicate differs from the target at any given position. Note the factor 1,000 in the scale of the z-axis.

Figure 11 shows the result for a number of duplicates *N* from 1 to 10 and for a divergence rate μ from 0 to 0.20. The surfaces have the same general aspect as in Figure 9. The probability that seeding is off-target increases with *N* and there is again a worst value of μ, because the maximum of $P\left({\bar{S}}_{0}\cap {\bar{S}}_{1}\right)$ minimizes expression (10) for every value of *N*. However, those values of μ are not the same (they are approximately 0.065 and 0.060 for skip-5 and skip-9 seeds, respectively).

Importantly, Figure 11 reveals that skipping 5 nucleotides increases the chances that seeding is off-target by a factor approximately 1.5, and skipping 9 nucleotides by a factor approximately 1.8 (compare with Figure 9). Skipping nucleotides seems to have little effect, but this is not a general conclusion. Indeed, skipping nucleotides decreases the probability of on-target seeding (skipping positions implies fewer on-target seeds) but increases the probability of null seeding (skipping positions implies fewer seeds overall), so the effects must be evaluated on a case-by-case basis. Skipping nucleotides can even decrease the probability that seeding is off-target. Concretely, for exact seeds of size γ = 19 in reads of size *k* = 50 with *N* = 10 duplicates at divergence μ = 0.1 with a sequencing error *p* = 0.1, the probability of off-target seeding is approximately 0.178 with exact seeds and 0.035 with skip-9 seeds, showing that skipping nucleotides can have different effects.

This kind of information is critical for choosing the best seeding strategy. Yet, the off-target seeding probability is not the only criterion. Equally important considerations are the probability of on-target seeding, the computational resources required to implement a particular seeding strategy and other sources of mapping errors (see discussion in section 8.1). The benefit of a theory to compute seeding probabilities is to have access to this knowledge.

## 6. Off-Target MEM Seeds

MEM seeds are substantially more complex than exact seeds and skip seeds because we need to take into account all the duplicates in the combinatorial construction.

### 6.1. Hard and Soft Masking

We first introduce two important notions that will be the key to understanding the behavior of MEM seeds.

**Definition 8**. *At a given position of the read, a duplicate is a hard mask if its match length on the left side is strictly longer than the match length of the target. A duplicate is a soft mask if it has the same match length as the target*.

**Figure 12:**
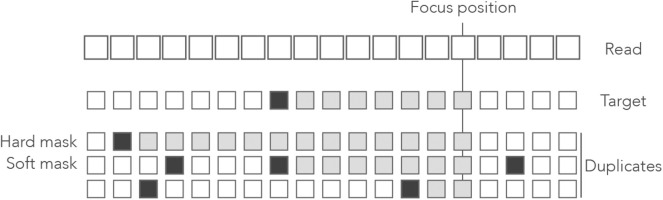
Example of hard and soft masks. Genomic sequences are shown below a read where open squares represent nucleotides. In the sequences, the open squares represent matches and the closed squares represent mismatches. The nucleotides contributing to the match length are represented as gray boxes. At the focus position, the match length of the target is 7. The first duplicate is a hard mask because its match length is 13 > 7. The second duplicate is a soft mask because its match length is 7, as the target. The third duplicate is not a mask because its match length is 2 < 7.

Figure 12 gives a graphical intuition of hard and soft masks. It is important to bear in mind that hard and soft masks depend on the position of interest: a sequence can be a mask at the left end of the read and not at the right end, or the opposite.

Hard and soft masks explain the counter-intuitive properties of MEM seeds. For instance, in Figure 4 the target cannot be discovered because every nucleotide of the read has a hard mask. In Figure 5, the target could be discovered if the read were shorter because a hard mask would turn into a soft one.

From the definition, we see that the last nucleotide of every strict on-target MEM seed is always unmasked. Conversely, an unmasked nucleotide always belongs to exactly one strict on-target MEM (not necessarily a seed because the size of the MEM can be less than γ). Also, the last nucleotide of every shared on-target MEM seed is always soft-masked, but a soft-masked nucleotide does not always belong to a shared on-target MEM.

Since hard and soft masks inform us about the positions of on-target MEM seeds, we construct an alphabet that encodes the masking status of the nucleotides.

### 6.2. The MEM Alphabet

As before, we recode the reads as sequences of letters from a specialized alphabet called the MEM alphabet $A=\{□,|,{↑}_{/1}\;,{↑}_{/2}\;,{↑}_{/3}\ldots ,{↓}_{/0},{↓}_{/1},{↓}_{/2},\ldots \}$.

The symbols ↓_/*m*_ (*m* ≥ 0) indicate that the nucleotide is a sequencing error and *m* is the number of duplicates that *match* the nucleotide. Since a sequencing error is always a mismatch against the target, the symbol ↓_/0_ indicates that the nucleotide is a mismatch against *every* sequence. The symbols ↑_/*i*_ indicate a change in masking status: the nucleotide is not masked but the previous is—this happens when all the masks fail to extend beyond this position. The index *i* ≥ 1 is the number of nucleotides since the last mismatch or since the beginning of the read. All the other nucleotides are represented by the symbol □, implying that □ symbols are never sequencing errors and always match the target. The symbol | is appended to the end of the read as before.

**Figure 13:**
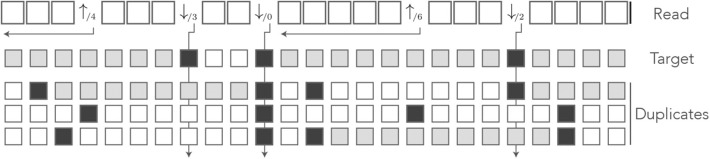
The MEM encoding. The read of Figure 3 is represented in the MEM alphabet. The arrows departing from the numbers help understand their meaning. The symbol ↓_/*m*_ is indexed by the number *m* of sequences that match the nucleotide. The symbol ↑_/*i*_ is indexed by the number *i* of nucleotides from the last error or from the beginning of the read. The gray squares in the symbolic sequences represent MEM seed matches. Other features are as in Figure 8.

Note that in the symbols ↓_/*m*_ and ↑_/*i*_, the numbers *m* and *i* have different meanings. In the symbol ↓_/*m*_, the index *m* is a number of sequences (0 ≤ *m* ≤ *N* where *N* is the number of duplicates); in the symbol ↑_/*i*_, the index *i* is a number of nucleotides. Figure 13 shows the encoding of a read in the MEM alphabet.

The MEM alphabet captures the masking status of the nucleotide: the symbol ↓_/*m*_ indicates that the nucleotide has *m* hard masks and *N* − *m* soft masks. The symbols ↑_/*i*_ indicate that the nucleotide is unmasked and that the previous nucleotide is masked.

In the MEM alphabet, strict on-target MEM seeds are the longest stretches of symbols containing some symbol ↑_/*i*_ and not containing any symbol ↓_/*m*_. Indeed, such a stretch is a match for the target because it does not contain any symbol ↓_/*m*_, it only matches the target because it contains at least one unmasked nucleotide (marked by ↑_/*i*_), and it cannot be extended because it is flanked by sequencing errors (symbols ↓_/*m*_) or by the ends of the reads. Note that there is exactly one symbol ↑_/*i*_ per strict on-target MEM seed, and therefore two symbols ↑_/*i*_ must be separated by at least one symbol ↓_/*m*_.

Shared on-target MEM seeds are the longest stretches of symbols □ flanked by ↓_/0_, or by the ends of the read. Indeed, such a stretch is a MEM seed because it matches the target and it cannot be extended (↓_/0_ is a mismatch against every sequence). Also, it cannot be a strict on-target MEM seed because it does not contain any ↑_/*i*_ symbol, so it must be a shared on-target MEM seed.

As before, the read is converted from a sequence of symbols to a sequence of segments that consist of 0 or more symbols □ followed by a terminator. We then specify the weighted generating functions of those segments and fill the transfer matrix $M_{N\left(z\right)}$ of the reads that do not contain an on-target MEM seed. We introduce the terms of the matrix by increasing order of complexity.

### 6.3. Segments Following ↑_/*i*_

A segment terminated by ↑_/*i*_ is the beginning of a strict on-target MEM of size at least *i*. The MEM reaches the next sequencing error or the end of the read, so the number of symbols □ in the next segment must be at most γ − *i* − 1 and it must be terminated by a ↓_/*m*_ symbol or by the tail terminator |.

The following definition will simplify the notations.

**Definition 9**. *Given the divergence rate μ and the number of duplicates N, the probability that a symbol is* ↓_/*m*_ *given that the nucleotide is a read error is*

(16) $$\omega_{m}=\left(\begin{array}{c} N\\ m \end{array}\right){\left(1-\mu /3\right)}^{N-m}{\left(\mu /3\right)}^{m}.$$

The expression for ω_*m*_ is exact: if the nucleotide is an error, the symbol is ↓_/*m*_ for some *m* between 0 and *N*. Each duplicate is a match with probability μ/3, so *m* has a Binomial distribution with parameters (*N*, μ/3).

On this segment, the matches between the read and the duplicates are irrelevant, so the weighted generating function of a symbol □ is simply *qz* (recall that *p* = 1 − *q* is the probability of a sequencing error). The weighted generating function of the terminator ↓_/*m*_ is ω_*m*_*pz*, so the weighted generating function of the ↓_/*m*_-segments following ↑_/*i*_ is

(17) $$D_{i,m}(z)=\omega_{m}pz\sum_{j=0}^{\gamma -i-1}{\left(qz\right)}^{j}.$$

And the weighted generating function of the tail segments following ↑_/*i*_ is.

(18) $$E_{i}(z)=\sum_{j=0}^{\gamma -i-1}{\left(qz\right)}^{j}.$$

### 6.4. Segments Following ↓_/*m*_

The symbol ↓_/*m*_ signifies that the nucleotide has *m* hard masks and *N* − *m* soft masks. If all the masks vanish before the first read error, the next terminator will be a symbol ↑_/*j*_, otherwise it will be the symbol | or a symbol ↓_/*m*_. We separate the cases based on the terminator of the segment.

#### Case 1: The Terminator Is ↑_/*j*_

**Definition 10**. *Given the divergence rate μ, the probability that a given duplicate contains a mismatch in a sequence of j error-free nucleotides is*

(19) $$\xi_{j}=1-{\left(1-\mu \right)}^{j}.$$

*This is the probability that a hard or soft mask vanishes within j correct nucleotides*.

The expression for ξ_*j*_ is exact: every nucleotide of the duplicate differs from the target with probability μ. In the absence of sequencing errors, this is also the probability that a nucleotide of the duplicate differs from the read. Given that there is no error, the probability that *j* nucleotides in a row are identical to the read is thus (1 − μ)^*j*^ and the probability that at least one of them is different is the complement 1 − (1 − μ)^*j*^.

With this notation, the probability that at least one of *N* masks survives a sequence of *j* error-free nucleotides is thus $1-{\left(\xi_{j}\right)}^{N}$, and the probability that there remains a mask at the *j* − 1-th but not at the *j*-th error-free nucleotide is ${\left(\xi_{j}\right)}^{N}-{\left(\xi_{j-1}\right)}^{N}$. From this we conclude that the weighted generating function of the segments terminated by ↑_/*j*_ following a segment terminated by ↓_/*m*_ is

(20) $$B_{j}(z)=({\left(\xi_{j}\right)}^{N}-{\left(\xi_{j-1}\right)}^{N}){\left(qz\right)}^{j}.$$

The fact that the reads have no on-target seeds imposes *j* < γ. Also note that this expression is the same for all symbols ↓_/*m*_ (it does not depend on *m*).

#### Case 2a: The Terminator | Comes Before the γ-th Nucleotide

In this case there can be no on-target seed because the read finishes too early. However, we must enforce the condition that at least one of the *N* masks survives until the end, otherwise the segment would be terminated by one of the symbols ↑_/*j*_. The weighted generating function is:

$$\sum_{i=0}^{\gamma -1}\left(1-{\left(\xi_{i}\right)}^{N}\right){\left(qz\right)}^{i}.$$

#### Case 2b: The Terminator | Comes After the γ-th Nucleotide

In this case, the soft masks do not hide the target. Even if a duplicate survives until the end of the read, there will be an on-target seed (shared in this case). To exclude on-target seeds, we must enforce the condition that at least one hard mask survives until the end of the segment (which is impossible if *m* = 0). The weighted generating function is

$$\sum_{i=\gamma }^{\infty }\left(1-{\left(\xi_{i}\right)}^{m}\right){\left(qz\right)}^{i}.$$

Summing the expressions from cases 2a and 2b, we find that the weighted generating function of the tail following ↓_/*m*_ is:

(21) $$C_{m}(z)=\sum_{i=0}^{\gamma -1}\left(1-{\left(\xi_{i}\right)}^{N}\right){\left(qz\right)}^{i}+\sum_{i=\gamma }^{\infty }\left(1-{\left(\xi_{i}\right)}^{m}\right){\left(qz\right)}^{i}.$$

#### Case 3a: The Terminator ↓_/*n*_ Comes Before the γ-th Nucleotide

In this case, there can be no on-target seed and we must only exclude the terminators ↑_/*j*_. As we have seen above, this implies that at least one of the *N* masks survives until the terminator. For a read of size *j* + 1, this occurs with probability $1-{\left(\xi_{j}\right)}^{N}$. Including the terminator and summing over *j* + 1 ≤ γ, we see that the weighted generating function is:

(22) $$\omega_{n}pz\sum_{j=0}^{\gamma -1}\left(1-{\left(\xi_{j}\right)}^{N}\right){\left(qz\right)}^{j}.$$

#### Case 3b: The Terminator ↓_/*n*_ Comes After the γ-th Nucleotide

This case is by far the most convoluted. Since the segment contains at least γ error-free nucleotides, we must enforce the condition that it does not contain an on-target seed. This will be the case if any of the two following conditions is validated: (i) at least one hard mask covers all the error-free nucleotides, or (ii) all the hard masks vanish but at least one soft mask covers the whole segment (including the terminator).

The two conditions are mutually exclusive by construction. They are graphically represented in the diagram below. The left panel corresponds to case (i) and the right panel to case (ii). The top row represents the target, and the bottom rows represent duplicates (using the same symbols as in Figure 13).


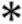


Whenever a hard mask (here the first duplicate) covers the nucleotides as shown by the gray squares in the left panel, there can be no on-target seed. The positions marked with a question mark are irrelevant, they cannot change the fact that there is no on-target MEM seed. If the hard masks vanish, as in the right panel, then we need to look at the soft masks. If a soft mask covers the whole segment as indicated by the gray squares, then there can be no on-target seed. In all other cases there is an on-target MEM seed.

For a segment of size *j* + 1, condition (i) has probability $\left(1-{\left(\xi_{j}\right)}^{m}\right)$. Summing over *j* + 1 > γ and including the terminator, we see that the associated weighted generating function is

$$\omega_{n}pz\sum_{j=\gamma }^{\infty }\left(1-{\left(\xi_{j}\right)}^{m}\right){\left(qz\right)}^{j}.$$

Condition (ii) is more convoluted, so we introduce some further notations to solve this sub-case.

**Definition 11**. *Given the divergence rate μ, the probability that a duplicate sequence contains a mismatch in a sequence of j error-free nucleotides followed by an error is*

(23) $$\eta_{j}=1-{\left(1-\mu \right)}^{j}\mu /3.$$

*This is the probability that a hard or soft mask vanishes within j correct nucleotides followed by a sequencing error*.

The expression for η_*j*_ is exact: the probability that there are *j* matches between the duplicate and the target is (1 − μ)^*j*^. If there are no sequencing errors, this is also the probability that there are *j* matches between the duplicate and the read. The probability that the duplicate matches the subsequent error is μ/3, so the probability that there are *j* + 1 matches including the sequencing error is (1 − μ)^*j*^μ/3. Finally, the probability that there is a mismatch is the complement 1 − (1 − μ)^*j*^μ/3.

Let us for now consider a segment of fixed size *j* + 1. From expressions (19) and (23), the probability of condition (*ii*) is

$${\left(\xi_{j}\right)}^{m}\left(1-{\left(\eta_{j}\right)}^{N-m}\right),$$

but we need to break up this term among all the possible terminators ↓_/*n*_ (0 ≤ *n* ≤ *N*) in order to fill the different entries of the transfer matrix. For this, we split this term in the number of soft masks that run until and including the terminator. From expression (23), the probability that there are *r* ≥ 1 such soft masks is

(24) $$\left(\begin{array}{c} N-m\\ r \end{array}\right){\left(1-\eta_{j}\right)}^{r}{\left(\eta_{j}\right)}^{N-m-r}.$$

For now we consider *r* fixed; we will compute the marginal probability at the final stage. By construction, each of those *r* soft masks matches the terminator, so the total number of matches is *r* plus the number of sequences that also match the terminator, among the remaining *N* − *m* − *r* soft masks and the *m* hard masks.

Let us start with the *m* hard masks. The probability that each of them matches the terminator is simply μ/3.

The case of the *N* − *m* − *r* soft masks is more complicated because they can vanish precisely on the terminator—recall that in case (*ii*) all the hard masks are assumed to vanish before. If the soft mask failed within the first *j* nucleotides, then the *j* + 1-th nucleotide can be anything and it will match the terminator with probability μ/3. But if the soft mask survived the first *j* nucleotides, then it *must* fail on the *j* + 1-th and it cannot match the terminator. From expressions (19) and (23), the probability that a given soft mask fails within the first *j* nucleotides is ξ_*j*_/η_*j*_—this is the conditional probability that it fails within the first *j* nucleotides given that it fails within the segment. Finally the probability that such a soft mask matches the terminator is μ/3 · ξ_*j*_/η_*j*_.

Summing the contributions of the hard masks (*m* in total, each matching the terminator with probability μ/3) and of the soft masks (*N* − *m* − *r* in total, each matching the terminator with probability μ/3·ξ_*j*_/η_*j*_), the probability that the total number of matches is *n* − *r* appears as the convolution product

$$\begin{array}{l} \frac{{\left(\mu /3\right)}^{n-r}{\left(1-\mu /3\right)}^{N-n}}{{\left(\eta_{j}\right)}^{N-m-r}}\psi_{j,m,n,r},\\ \text{ where }\psi_{j,m,n,r}=\sum_{q\geq 0}\left(\begin{array}{c} m\\ q \end{array}\right)\left(\begin{array}{c} N-m-r\\ n-r-q \end{array}\right){\left(\xi_{j}\right)}^{n-r-q}. \end{array}$$

Finally, we need to compute the marginal probability over the number *r* of soft masks that survive until the end of the read. Multiplying by the probability of *r* from expression (24) and summing over *r* ≥ 1, the probability that *n* duplicate sequences match the terminator appears as

$$\begin{array}{l} \;\sum_{r\geq 1}\left(\begin{array}{c} N-m\\ r \end{array}\right){\left(1-\eta_{j}\right)}^{r}{\left(\mu /3\right)}^{n-r}{\left(1-\mu /3\right)}^{N-n}\psi_{j,m,n,r}\\ ={\left(\mu /3\right)}^{n}{\left(1-\mu /3\right)}^{N-n}\sum_{r\geq 1}\left(\begin{array}{c} N-m\\ r \end{array}\right){\left(1-\mu \right)}^{rj}\psi_{j,m,n,r}\\ =\omega_{n}\cdot \zeta_{j,m,n}, \end{array}$$

where

(25) $$\zeta_{j,m,n}=\sum_{r\geq 1}\left(\begin{array}{c} N-m\\ r \end{array}\right){\left(1-\mu \right)}^{rj}\psi_{j,m,n,r}/\left(\begin{array}{c} N\\ n \end{array}\right).$$

This is the probability that the terminator is the symbol ↓_/*n*_ given that the segment has size *j* + 1 > γ, that the first sequencing error occurs on the last nucleotide, that the preceding terminator was ↓_/*m*_ and that the *m* hard masks fail before the end of the segment.

Summing the terms from case 3a and from case 3b, we find that the weighted generating function of the ↓_/*n*_ segments following ↓_/*m*_ is:

(26) $$\begin{array}{l} A_{m,n}\left(z\right)=\omega_{n}pz\sum_{j=0}^{\gamma -1}\left(1-{\left(\xi_{j}\right)}^{N}\right){\left(qz\right)}^{j}\\ \;+\omega_{n}pz\sum_{j=\gamma }^{\infty }\left(1-{\left(\xi_{j}\right)}^{m}\cdot \left(1-\zeta_{j,m,n}\right)\right){\left(qz\right)}^{j}. \end{array}$$

### 6.5. Transfer Matrix

Collecting and arranging the results above, we can verify that the final expression of the transfer matrix ${\overset{◦}{M}}_{N}\left(z\right)$ is

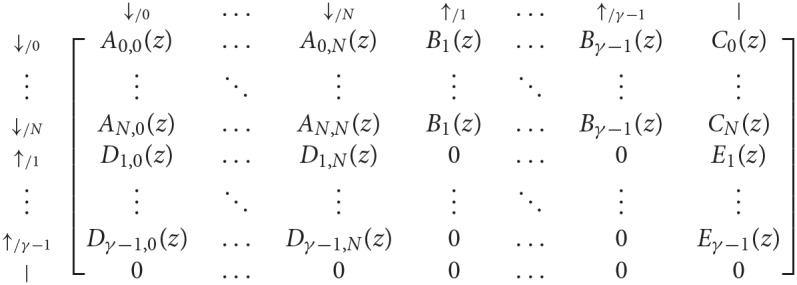



where

(26) $$\begin{array}{l} A_{m,n}\left(z\right)=\omega_{n}pz\sum_{i=0}^{\gamma -1}\left(1-{\left(\xi_{i}\right)}^{N}\right){\left(qz\right)}^{i}\\ \;+\omega_{n}pz\sum_{i=\gamma }^{\infty }\left(1-{\left(\xi_{i}\right)}^{m}\cdot \left(1-\zeta_{i,m,n}\right)\right){\left(qz\right)}^{i} \end{array}$$

(20) $$B_{i}(z)=\left({\left(\xi_{i}\right)}^{N}-{\left(\xi_{i-1}\right)}^{N}\right){\left(qz\right)}^{i}$$

(21) $$C_{m}(z)=\sum_{i=0}^{\gamma -1}\left(1-{\left(\xi_{i}\right)}^{N}\right){\left(qz\right)}^{i}+\sum_{i=\gamma }^{\infty }\left(1-{\left(\xi_{i}\right)}^{m}\right){\left(qz\right)}^{i}$$

(17) $$D_{j,m}(z)=\omega_{m}pz\sum_{i=0}^{\gamma -j-1}{\left(qz\right)}^{i}$$

(18) $$E_{j}(z)=\sum_{i=0}^{\gamma -j-1}{\left(qz\right)}^{i}$$

and where

(16) $$\omega_{m}=\left(\begin{array}{c} N\\ m \end{array}\right){\left(1-\mu /3\right)}^{N-m}{\left(\mu /3\right)}^{m}$$

(19) $$\xi_{j}=1-{\left(1-\mu \right)}^{j}$$

(23) $$\eta_{j}=1-{\left(1-\mu \right)}^{j}\mu /3$$

(25) $$\begin{array}{l} \zeta_{j,m,n}=\sum_{r\geq 1}\left(\begin{array}{c} N-m\\ r \end{array}\right){\left(1-\mu \right)}^{rj}\psi_{j,m,n,r}/\left(\begin{array}{c} N\\ n \end{array}\right)\\ \;\psi_{j,m,n,r}=\sum_{q\geq 0}\left(\begin{array}{c} m\\ q \end{array}\right)\left(\begin{array}{c} N-m-r\\ n-r-q \end{array}\right){\left(\xi_{j}\right)}^{n-r-q}. \end{array}$$

***Remark 4***. *In the special case N* = 0, *the transfer matrix simplifies to the extent that we can compute the weighted generating function of the reads without on-target MEM seed in closed form. The result is*

(2) $$\frac{1+qz+\ldots +{\left(qz\right)}^{\gamma -1}}{1-pz(1+qz+\ldots +{\left(qz\right)}^{\gamma -1})}.$$

*Expression (2) was shown in section 3.4 to be the weighted generating function of reads without on-target exact seed. This shows that when there are no duplicates, MEM seeds have exactly the same properties as exact seeds*.

### 6.6. Computing MEM Seeding Probabilities

The matrix ${\overset{◦}{M}}_{N}(z)\cdot {\left(I-{\overset{◦}{M}}_{N}(z)\right)}^{-1}$ contains the weighted generating functions of all the reads without on-target MEM seeds. The term of interest, as usual, is the top right entry. Indeed, every read can be prepended by ↓_/0_-segments and only by those, otherwise the read would start with fewer than *N* soft masks. Thus, reads without an on-target MEM seed are precisely the sequences of segments that can be appended to the symbol ↓_/0_ and that are terminated by a tail.

To compute this term, we proceed as in section 3.6, i.e., we compute the powers of ${\overset{◦}{M}}_{N}\left(z\right)$ in the arithmetic of truncated polynomials and we stop the iterations when the terms are negligible. We bound the probability that the read contains more than *e* sequencing errors using the expression

(7) $$Pr(X\geq e)\leq \exp\left((e-k)\log\frac{k-e}{k(1-p)}-e\log\frac{e}{kp}\right),$$

but here not every segment contains an error. There cannot be two symbols ↑_/*j*_ in a row, so a read with *s* + 1 segments must contain a minimum number of sequencing errors which is *e* = ⌊*s*/2⌋. As before, we compute the powers of ${\overset{◦}{M}}_{N}\left(z\right)$ until the upper bound is less than a set fraction ε of the current value of *a*_*k*_.

If we call *M*_0_ the event that the read contains an on-target MEM seed, the method above gives us $P\left({\bar{M}}_{0}\right)$. Calling *M*_*j*_ the event that the read contains a MEM seed for the *j*-th duplicate, we are interested in the probability

(8) $$P\left({\bar{M}}_{0}\cap (M_{1}\cup \ldots \cup M_{N})\right)=P({\bar{M}}_{0})-P\left({\bar{M}}_{0}\cap {\bar{M}}_{1}\cap \ldots \cap {\bar{M}}_{N}\right).$$

The key insight to compute $P\left({\bar{M}}_{0}\cap {\bar{M}}_{1}\cap \ldots \cap {\bar{M}}_{N}\right)$ is to realize that there is some MEM seed, on-target or not, if and only if the read contains a match of size γ or more for any of the *N* + 1 sequences. Therefore, this probability is the same as the term $P\left({\bar{S}}_{0}\cap {\bar{S}}_{1}\cap \ldots \cap {\bar{S}}_{N}\right)$ computed in section 4.

In conclusion, the probability that the MEM seeding process is off-target is

(27) $$P({\bar{M}}_{0})-P({\bar{S}}_{0})\cdot {\left(\frac{P({\bar{S}}_{0}\cap {\bar{S}}_{1})}{P({\bar{S}}_{0})}\right)}^{N},$$

where $P\left({\bar{M}}_{0}\right)$ is computed using ${\overset{◦}{M}}_{N}\left(z\right)$ as explained in this section, $P\left({\bar{S}}_{0}\right)$ is computed using a recursive equation as explained in section 3.4, and $P\left({\bar{S}}_{0}\cap {\bar{S}}_{1}\right)$ is computed using ${\tilde{M}}_{0}\left(z\right)$ as explained in section 4.1.

### 6.7. Monte Carlo Sampling

One potential difficulty in computing $P\left({\bar{M}}_{0}\right)$ is that the matrix ${\overset{◦}{M}}_{N}\left(z\right)$ has dimension (*N* + γ + 1) × (*N* + γ + 1). The problem can become computationally intractable because *N* can be very large. For instance, the sequences called *Alu* have more than one million duplicates in the human genome. There is no hope to compute the powers of ${\overset{◦}{M}}_{N}\left(z\right)$ in these conditions and we need an alternative method.

The symbolic representation as MEM segments can be used to design an efficient method to sample reads. Instead of generating the nucleotides of the *N* + 1 sequences one by one, we can generate a single sequence of segments. Since the number of segments does not depend on *N*, we can obtain a fast Monte Carlo method to sample millions of reads and count the proportion that contain an on-target MEM seed.

The principle is to proceed in cycles of two steps. We first sample the position of the next sequencing error, which gives the position of the next symbol ↓_/*m*_, where *m* will be determined at a later stage. The second step is to determine whether there is a symbol ↑_/*j*_ before that. For this we sample the number of masks that vanish before the symbol ↓_/*m*_. If they all vanish, the read contains an on-target MEM seed, provided the next read error is at a distance greater than γ. Otherwise, we sample the number *m* of hard masks at the sequencing error, and the process is repeated until we generate an on-target MEM seed, or until the read has size *k* or greater (in which case it has no on-target MEM seed).

The method is summarized in algorithm 1 below. It requires efficient algorithms to sample from the geometric and from the binomial distributions. Sampling from a geometric distribution can be done by computing the logarithm of a uniform (0, 1) random variable. Sampling from a binomial distribution can be done by the method of Kachitvichyanukul and Schmeiser (1988). Most importantly, the number of duplicates *N* has little influence on the running speed of algorithm 1.

**Parameter**: *k* is the size of the reads.

**Parameter**: *p* is the error rate of the sequencer (substitutions only).

**Parameter**: *N* is the number of duplicates.

**Parameter**: μ is the nucleotide-wise probability that duplicates differ.

**Result**: Sample a read at random. Return 1 if the read contains a good MEM seed, otherwise return 0.


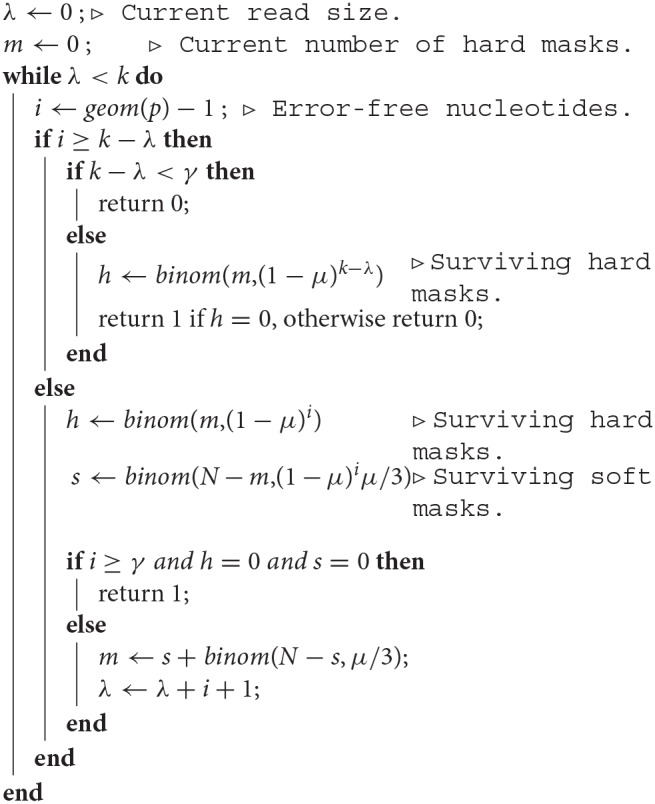


Algorithm 1 is an important result. It gives a compact solution to the problem of estimating the probability that a read can be mapped when using MEM seeds. The algorithm is also much faster than the naive approach of sampling every nucleotide of every sequence, because it is equivalent to sampling the nucleotide sequence of *all* the duplicates.

### 6.8. Illustration

We illustrate the strategy delineated above using the same settings as in section 4.6 (*k* = 50, *p* = 0.01, and γ = 19), except that we replace exact seeds by MEM seeds.

**Figure 14:**
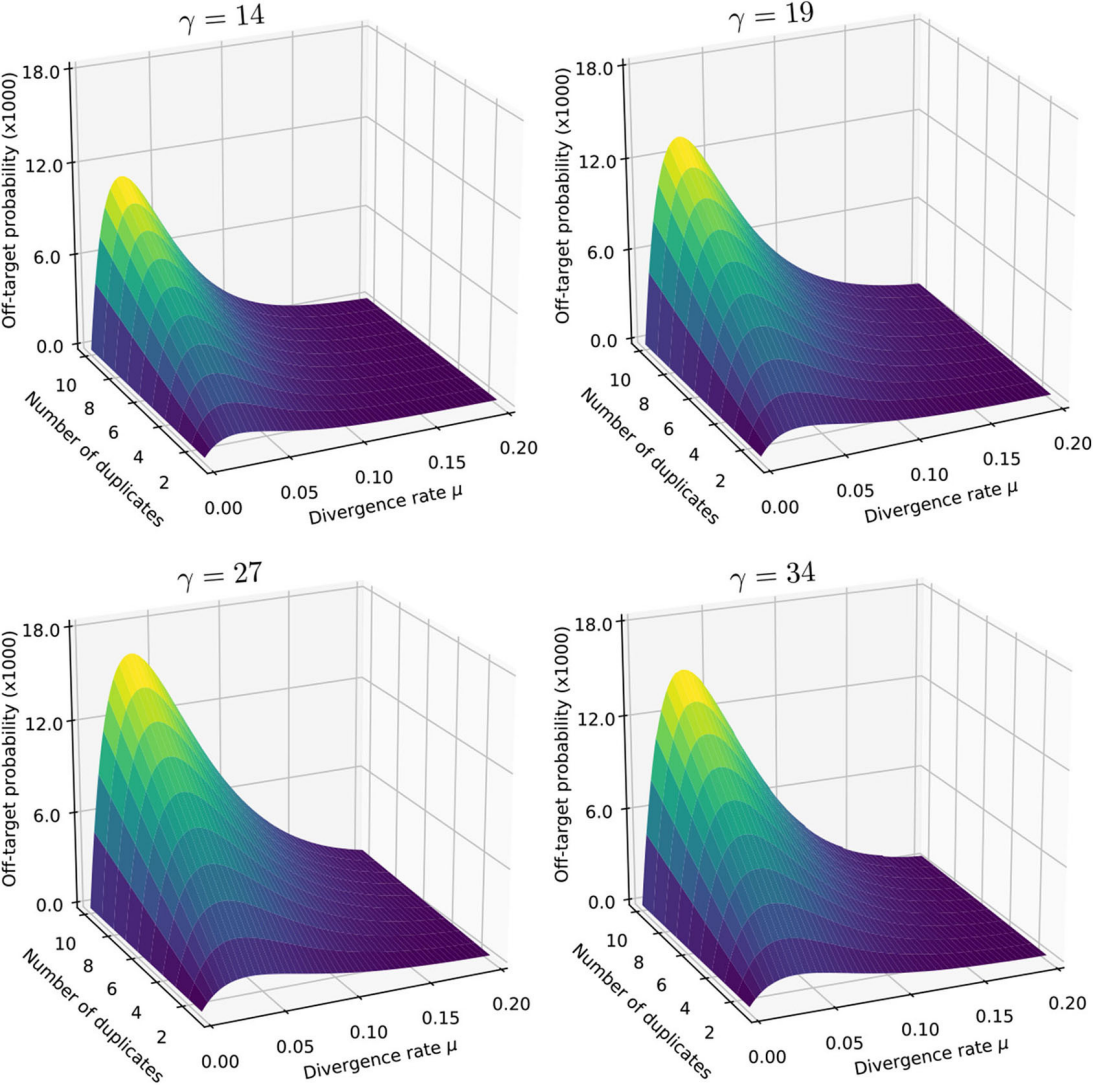
Off-target seeding probabilities (MEM seeds). The surfaces show the probability that seeding is off-target for MEM seeds of indicated minimum size γ. Read size and error rate are the same as in Figure 9, i.e., *k* = 50 and *p* = 0.01. The divergence rate μ is the probability that a given duplicate differs from the target at any given position. Note the factor 1,000 in the scale of the z-axis.

Figure 14 shows the result for a number of duplicates *N* from 1 to 10, for a divergence rate μ from 0 to 0.20 and for MEM seeds of different minimum size γ. The surfaces have the same general aspect as those of Figure 9. The probability that seeding is off-target increases with *N*, as shown by expression (27).

There is again a worst value of μ in each plot, but it is much lower than the previous two cases (it is close to 0.028 for γ = 14 and γ = 19, 0.030 for *gamma* = 27 and 0.035 for γ = 34). In the case of MEM seeds, it is not obvious why the same value of μ maximizes (27) for all values of *N* because both $P\left({\bar{M}}_{0}\right)$ and $P\left({\bar{S}}_{0}\cap {\bar{S}}_{1}\right)$ depend on μ.

Figure 14 reveals that in this concrete case, MEM seeds increase the chances that seeding is off-target by a factor 15 compared to exact seeds (see Figure 9). On this criterion, MEM seeds are always inferior to exact seeds with the same specifications. The reason is that a MEM seed always contains at least one exact seed of size γ. MEM seeds tremendously simplify the seeding process, but this comes at the cost of an increase of the probability that seeding is off-target.

Also note that the values change less than one might expect when varying the minimum seed length γ. Part of the reason is that MEM seeds are typically longer than their minimum size γ. In any event, this example shows that reducing γ to gain sensitivity provides very modest benefits, with potentially high costs in terms of short spurious hits.

## 7. The Sesame Library

We implemented the methods and algorithms presented here in an open-source C library to compute seeding probabilities. The library is called Sesame and is available at https://github.com/gui11aume/sesame.

### 7.1. Main Features of Sesame

Sesame contains functions to compute the seeding probabilities described here. All the functions were tested against simulations to ensure that the implementation is accurate and the code was checked extensively by static analysis and unit testing.

Computing seeding probabilities can take up to a few seconds, even when replacing iterative methods by Monte Carlo simulations. This is incompatible with the speed requirements of modern mappers, so Sesame has an interface for this type of application. In this mode, Sesame computes the results only the first time and stores them in memory for reuse on subsequent calls.

Storing the results in memory is an efficient strategy because three parameters are constant throughout the sequencing run: the minimum seed size γ, the read size *k* and the error rate of the sequencer *p* (and for skip seeds, the number of skipped nucleotides *n* is also constant). Only two parameters depend on the read: the number of duplicates *N* and their divergence rate μ. In this mode, Sesame automatically switches to Monte Carlo sampling when *N* is large to save time. Also, the input parameters are “snapped” to a predefined grid of set values for *N* and μ, so that few computations are performed and most of the calls are actually memory lookups. Sesame can thus be integrated in short read mappers without being a bottleneck.

Alternatively, the probabilities of interest can be computed offline, saved to disk and loaded at run time. This is particularly useful if the sequencing runs follow some standard conditions with a known error rate, because the computations can be recycled between runs.

Finally, Sesame also has an offline interface, where seeding probabilities are computed exactly as requested by the users, i.e., without modifying the algorithm or the parameters, and also without storing the results in memory.

The Sesame manual, available from the repository, contains additional information and explains in detail how to use the library.

### 7.2. Using Sesame to Compare Seeding Strategies

As a tool to compute seeding probabilities, Sesame can be used to compare the merits of different strategies. The kind of insight that we can gain from such calculations was already showcased in Figures 9, 11, 14, where the numbers were computed using Sesame.

To further showcase the potential benefits of computing seeding probabilities, we use Sesame to compare the default seeding strategies of BWA-MEM (Li, 2013) and Bowtie2 (Langmead and Salzberg, 2012). Note that both mappers use advanced techniques to refine the seeds, so this comparison does not reflect the true performance of the mappers. It is nevertheless useful to know the baseline of each strategy. The default of BWA-MEM is to use MEM seeds of minimum size 19; that of Bowtie2 is to use skip-9 seeds of size 16.

**Figure 15:**
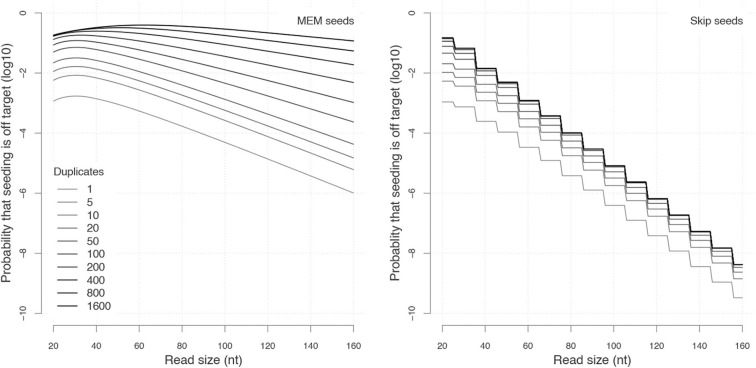
Comparing seeding strategies with Sesame. **(Left)** MEM seeds with γ = 19 and μ = 0.06. **(Right)** Skip-9 seeds with γ = 16 and μ = 0.06. The probabilities that seeding is off-target were computed using Sesame. Each curve represents the probability for a given number of duplicates (*N*). Estimates using iterative methods (MEM seeds where *N* ≤ 20) were computed to within 1% accuracy. Estimates using Monte Carlo sampling (MEM seeds where *N* > 20) were computed as the average of 500 million simulations.

The probabilities that seeding is off-target for different read sizes *k* and different number of duplicates *N* are plotted in Figure 15. The left panel shows the results for MEM seeds and the right panel shows the results for skip seeds. Here the error rate *p* is set to 1%, close to the specifications of the Illumina platform (Nakamura et al., 2011), and the divergence rate between duplicates μ is set to an arbitrary value of 6%.

The behaviors of the two types of seeds are dramatically different. Let us start with MEM seeds. For every value of *N*, the probability initially increases with the read size, and then drops exponentially. The initial increase is a hallmark of MEM seeds; it is due to the fact that duplicates can mask the target. Note that the asymptotic decay depends on the number of duplicates *N* because each duplicate can mask the target and thereby reduces the probability that it is discovered. Overalł, these results show that the performance of MEM seeds is poor when the target has more than approximately 20 duplicates.

Turning to skip seeds, we see that the curves have a staircase look with a drop every 10 nucleotides. This is so because seeding probabilities remain unchanged until there is space for another seed of size 16 on the read. The curves are otherwise decreasing with a steady exponential trend where the asymptotic decay does not depend on the number of duplicates *N*. The reason is that duplicates do not prevent the target from being discovered, they merely fool the mapper when the target was not found. This property makes the asymptotic decay of skip seeds substantially faster that of MEM seeds when *N* is large.

Those properties are self-evident in retrospect, but they are not necessarily obvious from the definitions of MEM seeds and skip seeds. The main limitations of the MEM seeds are best understood by keeping in mind that their asymptotic decay depends on *N*.

Figure 15 suggests that skip-9 seeds of size 16 are just better than MEM seeds of size 19. However, the gain in sensitivity comes at the cost of a larger candidate set, slowing down the mapping process. To remain competitive, Bowtie2 further filters the candidate set using a priority policy. But since some candidates are not checked, the probability that seeding is off-target is larger than shown in Figure 15. Also, we will show in section 8.1 that the higher sensitivity of skip seeds is not as big an advantage as it looks because seeding is not the only source of mapping errors.

### 7.3. Key Insights About MEM Seeds

At least two key insights about MEM seeds can be gained from Figure 15. The first is that it is not worth it for a MEM-based mapper to check all the candidate loci when there are more than approximately 20 of them. The mapper may find the correct location, but even if this is the case, the mapping quality will remain low because the prior chances of failure were high. A better strategy is to either bail out to not waste time, or to switch to a more sensitive seeding method (BWA opts for the second and uses a re-seeding policy). It is also important to note that this decision should be based on an estimated value of *N* and not, for instance, on the size of the seed or some other variable.

A second insight is that for MEM seeds, the off-target rate is always above 10^−3^ for reads of 50 nucleotides or fewer. Here it is important to mention that the value μ = 0.06 is not even the worst for reads of this size when *p* = 0.01 (according to Figure 14, the worst value is around 0.02–0.03). So if μ is unknown and one wishes to be conservative, it seems that reads of 50 nucleotides cannot be mapped with confidence better than 1/1, 000.

However, this is not true for the reason that it is practically impossible to map an Illumina read of size 50 to the wrong location when *N* = 0 (see section 8.3). Indeed, incorrect locations are unrelated sequences in this case, and it is easy to recognize that two sequences of size 50 are not homologous. This means that the highest impact one can have on the mapping quality is to check whether *N* is 0, or in other words, whether the target is a unique sequence.

These insights suggest that there is a way to make MEM seeds more useful for short read mappers. Following these principles, we have implemented a prototype mapper based on Sesame that shows good overall performance (Zorita et al., 2020).

## 8. Practical Considerations

### 8.1. True vs. Best Location

Early in the development of the theory, we distinguished the true location from the best location. We swiftly assumed that they are identical in order to eliminate some practical considerations that would otherwise clutter the exposition. Throughout the article we have developed a framework to compute the probability that the *true* location is in the candidate set. We have not mentioned anything about the probability that the *best* location is in the candidate set, so our results do not address the best location problem. It remains to establish how they contribute to addressing the *true* location problem.

The issue at hand is that the candidates are tested with an alignment algorithm that returns the best location. So even if the true location is in the candidate set, the read may be mapped somewhere else because some other candidate has a better alignment score. The question is how often this happens. If this is a rare event, our results give a good approximation of the probability that a read is mapped to the true location. Otherwise the estimates may not be so useful in practice.

The answer depends on the seed type. We start with MEM seeds because they will allow us to highlight an important phenomenon. The key insight is that MEM seeds tend to exclude the target from the candidate set if it is not the best location. To develop an intuition as to why this is the case, consider a read with a single sequencing error. The only way a candidate can have a better score is to be a perfect match for the read. But in this case the true location is hard-masked (see definition 8) and it is not in the candidate set.

Excluding ties, there are two possibilities: either the true location is the best, in which case it is seeded and the read is correctly mapped; or the true location is not the best, in which case it is not seeded and the read is mapped incorrectly. The read is mapped to the true location if and only if seeding is on-target. In practice there are ties, and reads can have more than one error, but this is a general trend for MEM seeds.

Table 1 shows the results of simulations with MEM seeds where we dissociate the true from the best location. In each tested condition, we record how often the reads are mapped incorrectly because seeding was off-target. Importantly, we also record the cases where reads are mapped incorrectly despite the fact that seeding was on-target. The results show that the second case occurs a minor fraction of the time, meaning that the probability that seeding is on-target is close to the probability that the read is mapped to the true location.

**Table 1:** Simulations of mapping errors with MEM seeds.

***k***	***N***	**Not seeded**	**Seeded, not best**	**Relative error**
50	1	9.2·10^−4^	8.4·10^−5^	1.09
50	10	9.0·10^−3^	7.9·10^−4^	1.09
50	100	8.1 · 10^−2^	5.1 · 10^−3^	1.06
100	1	2.4 · 10^−5^	7.3 · 10^−6^	1.30
100	10	2.9 · 10^−4^	6.9 · 10^−5^	1.24
100	100	5.5 · 10^−3^	5.7 · 10^−4^	1.10

*Ideal reads were randomly generated 1,000,000 times with sequencing error rate p = 0.01, divergence rate μ = 0.06, varying the read size k and number N of duplicates as indicated, using MEM seeds of minimum size γ = 19. Not seeded shows how often the read had no seed for the target, Seeded, not best shows how often the read had a seed for the target but the target was not the best candidate, and Relative error shows the factor between the probability that the read is not mapped to the true location and the probability that seeding is off-target*.

For exact and skip seeds, the conclusions are different. The situation is even the opposite if we consider reads with a single sequencing error. In this case the target is always in the candidate set (if the read size is greater than 2γ, where γ is the minimum seed length), so mapping errors are never due to failures of the seeding heuristic. Instead, mapping errors happen only when the true location is not the best.

Table 2 shows the results of similar simulations with skip-9 seeds. In all the tested cases the probability that the true location is not the best is substantially higher than the probability that seeding is off-target. As a result, the Sesame estimates are very far from the probability that the read is not mapped to the true location. The results for exact seeds are omitted, but they are qualitatively similar to those obtained for skip-9 seeds.

**Table 2:** Simulations of mapping errors with MEM seeds.

**k**	***N***	**Not seeded**	**Seeded, not best**	**Relative error**
50	1	1.7 · 10^−4^	4.9 · 10^−4^	3.9
50	10	1.6 · 10^−3^	4.7 · 10^−3^	3.9
50	100	7.9 · 10^−3^	4.4 · 10^−2^	6.6
100	1	4.4 · 10^−8^	2.3 · 10^−5^	523.7
100	10	3.5 · 10^−7^	2.3 · 10^−4^	658.1
100	100	1.0 · 10^−6^	2.3 · 10^−3^	2301.0

*Ideal reads were randomly generated 1,000,000 times with sequencing error rate p = 0.01, divergence rate μ = 0.06, varying the read size k and number N of duplicates as indicated, using skip-9 seeds of size γ = 19. The meanings of the columns are the same as in Table 1*.

In conclusion, the off-target probabilities computed by Sesame are close to the probability that the read is not mapped to the true location when using MEM seeds. In contrast, the estimates are very far when using exact seeds and skip seeds. In this case, it is more accurate to use an estimate of the probability that the true location is not the best, which is relatively easy to compute given the error rate of the sequencer *p*, the number of duplicates *N* and their divergence rate μ.

### 8.2. Realistic Sequencing Errors

In order to make the model tractable, we had to make some simplifying assumptions regarding the distribution of errors (section 3.1). In particular, we assumed that sequencing errors are uniform on the read, which is known to not be the case with current instruments. The errors are typically more frequent at the ends of the reads (Nakamura et al., 2011), meaning that seeds may be longer than expected, impacting the probability that seeding is off-target.

To test whether this is the case, we used the ART simulator (Huang et al., 2012) to emulate the error distribution of Illumina reads as per technology standards of 2016. We used the settings for the Illumina HiSeq 2000 and generated random reads of size 50 and 100 from the human genome. We estimated the probability that seeding is off-target from simulations and compared the results to calculations performed by Sesame (ART suggests error rates *p* = 0.0052 and *p* = 0.0075 for reads of size 50 and 100, respectively).

Table 3 shows the results for MEM seeds of minimum size γ = 19. The discrepancy with Sesame estimates is always within a factor 1.2. Table 4 shows the same type of comparison for skip-9 seeds of size γ = 19. The values are substantially lower than for MEM seeds because skip-9 seeds are more sensitive. In these conditions, the discrepancy with the Sesame estimates is within a factor 1.5, but it is important to highlight that simulations are unreliable for events with very low probability.

**Table 3:** Simulations of seeding errors with MEM seeds.

**k**	***N***	**Sesame**	**Simulation**	**Ratio**
50	1	4.5 · 10^−4^	3.8 · 10^−4^	1.18
50	10	4.5 · 10^−3^	4.4 · 10^−3^	1.02
50	100	4.0 · 10^−2^	4.2 · 10^−2^	0.95
100	1	3.7 · 10^−5^	3.5 · 10^−5^	1.05
100	10	4.2 · 10^−4^	4.1 · 10^−4^	1.02
100	100	8.9 · 10^−3^	8.6 · 10^−3^	1.03

*Realistic Illumina HiSeq 2,000 reads were randomly generated 1,000,000 times varying the read size k and number N of duplicates as indicated, and fixing the divergence rate to μ = 0.06 and using MEM seeds of minimum size γ = 19. Sesame shows the probability that seeding is off-target as computed with Sesame, Simulation shows this probability estimated by the simulation, and Ratio shows their ratio*.

**Table 4:** Simulations of seeding errors with skip-9 seeds.

**k**	***N***	**Sesame**	**Simulation**	**Ratio**
50	1	4.5 · 10^−5^	4.5 · 10^−5^	1.00
50	10	4.2 · 10^−4^	3.9 · 10^−4^	1.07
50	100	2.1 · 10^−3^	1.9 · 10^−3^	1.11
100	1	<10^−6^	<10^−6^	NA
100	10	3 · 10^−6^	2 · 10^−5^	1.50
100	100	8 · 10^−6^	6 · 10^−4^	1.33

*Realistic Illumina HiSeq 2,000 reads were randomly generated 1,000,000 times varying the read size k and number N of duplicates as indicated, and fixing the divergence rate to μ = 0.06 and using MEM seeds of minimum size γ = 19. The meanings of the columns are the same as in Table 3*.

Overall, these results suggest that the relatively strong assumptions regarding the distribution of errors are not a major issue in practice. The answer of course depends on the type of sequencer that is used and on the particulars of the mapping problem.

In practical applications, it is likely that other factors will be more detrimental for the precision of the estimates. For instance, the number of duplicates *N* and their divergence rate μ are unknown, introducing an uncertainty that propagates to the Sesame estimates. Importantly, we assumed that duplicates evolve independently of each other and with uniform substitutions even in the simulations featured in Tables 3, 4 because more realistic models are tedious to implement. The burden of this assumption on the precision of the estimate in practical settings is difficult to gauge. Overall, Sesame estimates should be considered approximate in all applications with real biological data.

### 8.3. Spurious Random Hits

We have assumed that seeds can match only the target or one of its duplicates. In reality, seeds have a small chance to match any sequence of the genome.

We have assumed throughout that all the genomic sequences that have a perfect match for a seed are considered candidate locations. The hope is that spurious random matches are shorter than the minimum seed length γ, but this is not always the case. And as mentioned above, every candidate location must be verified by an exact alignment algorithm.

Such random hits can be problematic for two reasons: First, the time spent verifying them is wasted. Second, they can cause false positives. Fortunately, both issues can be addressed.

To save time during the alignment phase, we can prioritize the seeds so that the best location is likely to be discovered first, allowing us to bail out from other alignments as early as possible. In some cases, it is even possible to give an upper bound on the alignment score of a candidate location so that the alignment can be skipped altogether. These considerations depend on the implementation of the mapper so we will not develop this further. What matters is that random hits do not impose a significant burden on the time needed to verify the candidates.

The second issue is that random hits can generate false positives. Such cases occur when seeding is null (meaning that there is no seed for either the target or any of its duplicates). Even when seeding is null, the candidate set is in general not empty because of spurious random hits. But since such hits are not homologous to the read, the alignment score is noticeably low, and this case is easy to detect.

Indeed, if the candidate location is random, mismatches occur with probability 3/4. If it is the true location, mismatches occur with probability *p*. We discard the seed because it is an automatic match of size γ (and possibly larger in the case of MEM seeds). Say that there remain *L* nucleotides and that *m* of them are mismatches for the candidate location. From Bayes formula, the probability that the location is random given the number of mismatches is

(28) $$\frac{1}{1+4^{L}{\left(p/3\right)}^{m}q^{L-m}(1-\beta )/\beta },$$

where *q* = 1 − *p* and β is the prior probability that the hit is spurious.

The value of β has little importance if *p* is small. To give an example, say that *p* = 0.01 and that a read generates a hit in the genome such that 33 nucleotides need to be aligned after seeding. If the hit is random there is a 99.99% chance that at least 15 are mismatches. For *m* = 15, the denominator of expression (28) is approximately 1 + 4.3 · 10^−18^(1 − β)/β. So unless β < 10^−17^, the support for the hypothesis that the hit is random is overwhelming. Conversely, if the hit is not random, there is a 99.99% chance that 4 or fewer nucleotides are mismatches. For *m* = 4, the denominator is approximately 1 + 6.8 · 10^9^(1 − β)/β, so unless 1 − β < 10^−9^, the support for the hypothesis that the hit is not random is overwhelming. In summary, if *p* is small, we do not need to worry about the value of β, one can choose for instance β = 1/2 so that the term (1 − β)/β disappears from expression (28).

Since the probability that the best candidate is a random sequence is either very small or very large, it has no influence in the first case, and it dominates the probability of a false positive in the second case. Checking the value of expression (28) after mapping reveals whether the hit should be discarded and the read should be considered unmapped.

In summary, spurious random hits occur regularly, but it is possible to minimize their computational burden. Also, they are no cause for concern regarding false positives because they can easily be detected using Bayes' formula, as shown in expression (28).

## 9. Discussion

We have devised a set of methods to compute the probability that seeding heuristics fail and commit the mapping process to an error. We have also implemented the algorithms as an open source C library to perform the computations. This fills a knowledge gap to understand and calibrate the performance of the seeding heuristics. The pillars of our strategy are borrowed from analytic combinatorics (Régnier, 2000; Nicodeme et al., 2002; Flajolet and Sedgewick, 2009; Sedgewick and Flajolet, 2013), even though we do not follow the complete programme. Constructing generating functions usually serves the purpose of finding their singularities in order to approximate the solution. In our case, however, the weighted generating functions cannot be computed so this strategy is not applicable.

To find the probabilities of interest in the absence of a fully specified weighted generating function, we compute only the first terms of the Taylor series using iterative methods as explained in section 3.6, or using Monte Carlo sampling as explained in section 6.7. In this regard, the breakthrough is the encoding of reads as segments in different alphabets, which is an implicit form of Markov imbedding (Fu and Koutras, 1994).

Our strategy relies on the knowledge of two essential parameters: the number of duplicates *N* and their divergence rate μ. These quantities can be estimated efficiently using the FM-index (Ferragina and Manzini, 2005) as shown in our related work on a prototype mapper based on the concepts developed in this article (Zorita et al., 2020).

The method presented here is general, but it is important to clearly state the assumptions it depends on. First and most importantly, we have ignored insertions and deletions. We assume that the sequencing errors are substitutions only, which makes the method adapted to the Illumina technology, but not to deletion-prone instruments such as the Oxford Nanopore technology. We also assume that insertions and deletions never occur among duplicated sequences. This is obviously incorrect, but our initial tests with real data suggest that this is a minor impediment. Incorporating insertions and deletions would make the theory intractable, so it is presently unclear how to deal with this type of error.

The second assumption is that the candidate set consists of all the genomic locations that have a perfect match for at least one seed, and that all the elements of the candidate set are tested with an exact sequence alignment algorithm. This is possible, but it is important to note that for plant and animal genomes, a single seed may have tens of thousands of hits. Therefore, most mappers impose a limit on the number of alignments per read, opening the possibility that the best hit is seeded but not aligned. It is clear that in this case, the probability that the target is in the candidate set has little to do with the probability that the read is mapped correctly. This is again a minor impediment, since the probability of mapping such reads correctly is low either way. Mappers can have a lower range of confidence score when the candidate set is too large to check every sequence.

The third assumption is that all the duplicate sequences evolve independently of each other and at the same rate. This is again incorrect because duplication events can happen continuously, creating complex ancestry relationships. It is possible to infer the ancestry using tree reconstruction techniques, but it would be challenging to incorporate this information in the present theory. The symbols of the alphabets developed above implicitly assume that the sequences are exchangeable and the complexity of the calculations explodes if it is not the case.

The last assumption is that seeds can match only the target or its duplicates. This does not hold in general because the candidate set usually contains spurious random hits, but we have shown how to deal with this possibility *a posteriori* in section 8.3.

We have not computed off-target probabilities for spaced seeds. In section 3.5 we highlighted the general strategy to compute on-target probabilities with our approach. Traditionally, such on-target probabilities have been computed using finite automata (Buhler et al., 2005; Kucherov et al., 2005), with the caveat that such automata can contain a very large amount of states. There is thus an interest in developing methods to reduce the number of states so that computations can be performed fast (Martin and Noé, 2017). Interestingly, the method presented in section 3.5 generates 2^*m*^ + 1 states (this is the size of the transfer matrix), where *m* is the number of don't-care positions in the seed model. This is typically lower than the number of states in the automata, but proper benchmarks would be required to know if our method brings real benefits.

Computing the off-target probabilities for spaced seeds can be done with the strategy presented in section 4, but this brings the dimension of the transfer matrix to 4^*m*^ + 1. To give a concrete idea, the seed of PatternHunter (Ma et al., 2002) has 7 don't-care positions, meaning that the transfer matrix would have 16,385 rows and columns. Even though the matrix is sparse, the computation time can be expected to be prohibitive.

Being able to compute seeding probabilities revealed some interesting facts (sections 4.6, 5.7, and 6.8). The first is that the seeding schemes considered here have a worst case scenario for a particular value of μ, the divergence between duplicates. Importantly, the worst value varies between different seeding methods, so it is possible in theory to construct opportunistic seeding strategies that pick the best method for every read, depending on the value of μ. Another interesting fact is that skip seeds can have better performance than exact seeds in the sense that they can yield lower off-seeding probabilities (section 5.7). However, this always comes at the cost of accuracy because skipping nucleotides reduces the probability of on-target seeding.

We also observed that MEM seeds have a significantly higher off-target seeding rate compared to exact seeds and skip seeds (section 6.8). This does not mean that MEM seeding is a bad strategy (it is usually faster than the other methods), but it is good practice to keep an eye on performance and switch methods or even skip the read altogether if the chances of discovering the target are too low. We also showed in section 8.1 that for MEM seeds, the on-target seeding rate is close to the probability that the read is mapped to the true location, which was not the case for exact seeds and skip seeds. In this regard, the present theory is most useful when using MEM seeds.

Regarding the methodology, the Monte Carlo approach of algorithm 1 is relatively straightforward, so one may wonder why the approach with weighted generating functions would be necessary at all for MEM seeds. The only reason is precision. To estimate the frequency of an event by Monte Carlo sampling, this event must occur at least a few times in the simulation. For instance, with 1 million rounds of sampling, frequencies around 1/100, 000 or lower cannot be measured accurately. When one is interested only in frequent events, it is thus a reasonable strategy. On the other hand, for *N* < 20, the probability that MEM seeding is null or off-target is relatively small, so we need a method that is accurate in this range. Fortunately, the transfer matrix method is fast because the dimension of the matrix ${\overset{◦}{M}}_{N}\left(z\right)$ is small and the computations are not prohibitive for small values of *N*.

The proposed methods meet the demand for speed. One needs to compute the probabilities only once for a given value of *N* and μ (the error rate *p* is known and constant). For *N* > 20, the iterative method is usually too slow and we need to use Monte Carlo sampling instead. The running time depends on *p*, on the size of the reads *k*, and on the desired number of iterations. Since those are constant throughout the sequencing run, the method always takes the same amount of time (around 1-10 seconds for 1,000,000 simulations of reads of size around 100 nucleotides on modern hardware). The values of *N* and μ can be binned in intervals so that there are only around 100 pairs for a total cost of a few minutes per run. Considering that mappers seem to process at most 10, 000 reads per second per core, the time of mapping a sequencing run of 250 million reads is over 7 h per core, two orders of magnitude larger than the time required to estimate the probabilities of error.

Finally, one may wonder if our approach has any advantage over methods based on machine learning. Such algorithms have already proved useful (Lee et al., 2014) and the rapid progress in the field of deep learning suggests that it is possible to train algorithms to accurately estimate mapping quality. In time, such algorithms may prove faster and/or more robust because they could learn intrinsic biases of the mapping algorithms. Yet, the main benefit of our approach will remain: the combinatorial constructions are a direct access to the nature of the problem. For instance, viewing MEM seeds through the lens of hard and soft masks turns a seemingly intractable process into a relatively simple one (see algorithm 1). The combinatorial stance is that there is value in the models themselves.

In conclusion, we presented a practical solution to the problem of estimating the probability of false positives when using seeding heuristics. This solution is adapted for mapping short reads sequenced with the Illumina technology. Being able to calibrate the seeding heuristic not only allows the user to choose how to balance speed versus accuracy, but also opens new applications. For instance, one can map reads from contaminated samples in pools of closely related genomes (e.g., modern human and Neanderthal) in order to assign the reads to the organism they belong to. In this case, the probabilities of false positives give the right level of confidence in the assignment.

More generally, the analytic combinatorics programme is a very powerful tool to address problems in bioinformatics. Here we have seen how this strategy can be useful even when the generating functions cannot be computed. Using the same ideas, one could calibrate heuristics used in other alignment methods, especially in the expanding field of long-read technologies.

## Data Availability Statement

All datasets generated for this study are included in the manuscript/Supplementary Files.

## Author Contributions

GF designed the theory, wrote the C library, and wrote the manuscript. RC and EZ contributed to the theory and edited the manuscript.

## Conflict of Interest

The authors declare that the research was conducted in the absence of any commercial or financial relationships that could be construed as a potential conflict of interest.
