# Supplementary material for: Calibrating Seed-Based Heuristics to Map Short Reads With Sesame
Source: Front Genet. 2020 Jun 25;11:572. doi: 10.3389/fgene.2020.00572 (PMC7331467; doi:10.3389/fgene.2020.00572)
Supplement: Supplementary file 1 [file Presentation_1.zip › Appendix.pdf]

Calibrating seed-based heuristics to map short reads with Sesame: appendix

A Definitions

For convenience we reproduce here the definitions introduced in the text.

Definition 1. *The target is the DNA fragment that was actually sequenced. Duplicates are sequences of the genome that share homology with the target (in genetics they are often referred to as paralogs). In this article we will focus on short reads from complex eukaryotic genomes, so for concreteness the reader can assume that fragments are 30-300 bp long and that duplicates have above 75% identity with the target.*

Definition 2. *The output of the seeding step is the candidate set. The candidate set is the list of genomic locations where the read can be potentially mapped. The read is always mapped to one element of the candidate set. The seeding step is said to be*

i) "on target" if the candidate set contains the target,

ii) "off-target" if the candidate set contains a duplicate but not the target,

iii) "null" if the candidate set contains neither.

In this article, we will always consider that a genomic location is in the candidate set if and only if the read contains at least one seed with a perfect match for this genomic location.

Definition 3. *A seed is a subsequence of the read that has size at least γ (defined by the context of the problem) and that has at least one perfect match in the reference genome. Every genomic match of every seed is in the candidate set.*

Definition 4. *A Maximal Exact Match (MEM) is a subsequence of the read that is present in the reference genome and that cannot be extended—either because the read ends or because the extended subsequence is not in the genome.*

Definition 5. *A* strict *MEM seed has a single match in the genome. A* shared *MEM seed has several matches in the genome.*

Definition 6. *Let $\mathcal{A}$ be a set of combinatorial objects such that $a \in \mathcal{A}$ has a size $|a| \in \mathbb{N}$ and a weight $w(a) \in \mathbb{R}^+$. The weighted generating function of $\mathcal{A}$ is defined as*

$$A(z) = \sum_{a \in \mathcal{A}} w(a) z^{|a|}, \tag{1}$$

Expression (1) also defines a sequence $(a_k)_{k\geq 0}$ *such that*

$$A(z)=\sum_{k=0}^{\infty} a_k z^k.$$

By definition $a_k=\sum_{a\in A_k} w(a)$*, where* A_k *is the class of objects of size* k *in* $\mathcal{A}$*. The number* a_k *is the total weight of objects of size* k*.*

Definition 7. *A terminator is any symbol that is different from the symbol* $\Box$*. A segment is a sequence of 0 or more* $\Box$ *symbols followed by a terminator. The tail is the last segment of the read, where the terminator is always the special symbol* $|$*.*

Definition 8. *At a given position of the read, a duplicate is a* hard mask *if its match length on the left side is strictly longer than the match length of the target. A duplicate is a* soft mask *if it has the same match length as the target.*

Definition 9. *Given the divergence rate* μ *and the number of duplicates* N*, the probability that a symbol is* $\downarrow_{/m}$ *given that the nucleotide is a read error is*

$$\omega_m=\binom{N}{m}\big(1-\mu/3\big)^{N-m}\big(\mu/3\big)^{m}. \tag{16}$$

Definition 10. *Given the divergence rate* μ*, the probability that a given duplicate contains a mismatch in a sequence of* j *error-free nucleotides is*

$$\xi_j=1-(1-\mu)^j. \tag{19}$$

This is the probability that a hard or soft mask vanishes within j *correct nucleotides.*

Definition 11. *Given the divergence rate* μ*, the probability that a duplicate sequence contains a mismatch in a sequence of* j *error-free nucleotides followed by an error is*

$$\eta_j=1-(1-\mu)^j\mu/3. \tag{23}$$

This is the probability that a hard or soft mask vanishes within j *correct nucleotides followed by a sequencing error.*

B Complements

B.1 Derivation of expression (2)

In section 3.4, we had to compute the top right entry of the matrix $M_*(z)$ defined as $M_*(z)=M_0(z)+M_0(z)^2+\ldots=M_0(z)\cdot(I-M_0(z))^{-1}$, where

$$M_0(z)=\begin{bmatrix}\big(1+qz+\ldots+(qz)^{\gamma-1}\big)pz & 1+qz+\ldots+(qz)^{\gamma-1}\\ 0 & 0\end{bmatrix}.$$

Since $I-M_0(z)$ is a 2×2 matrix, we can invert it analytically. Observing that the determinant of $I-M_0(z)$ is $\Delta(z)=1-\big(1+qz+\ldots+(qz)^{\gamma-1}\big)pz$, we find

$$(I-M_0(z))^{-1}=\frac{1}{\Delta(z)}\begin{bmatrix}1 & 1+qz+\ldots+(qz)^{\gamma-1}\\ 0 & 1-\big(1+qz+\ldots+(qz)^{\gamma-1}\big)pz\end{bmatrix}.$$

Premultiplying by $M_0(z)$, we finally obtain

$$M_*(z)=\frac{1}{\Delta(z)}\begin{bmatrix}\big(1+qz+\ldots+(qz)^{\gamma-1}\big)pz & 1+qz+\ldots+(qz)^{\gamma-1}\\ 0 & 0\end{bmatrix},$$

where the top right term is equal to

$$\frac{1+qz+\ldots+(qz)^{\gamma-1}}{1-\big(1+qz+\ldots+(qz)^{\gamma-1}\big)pz}. \tag{2}$$

B.2 Derivation of expression (3)

In section 3.4, we had to extract the Taylor coefficients $a_0, a_1, a_2\ldots$ of the function

$$\frac{1+qz+\ldots+(qz)^{\gamma-1}}{1-\big(1+qz+\ldots+(qz)^{\gamma-1}\big)pz}=a_0+a_1z+a_2z^2+\ldots$$

Multipying both terms of the equation by $1-pz\big(1+qz+\ldots+(qz)^{\gamma-1}\big)$ we obtain

$$1+qz+\ldots+(qz)^{\gamma-1}=\big(1-\big(1+qz+\ldots+(qz)^{\gamma-1}\big)pz\big)\,(a_0+a_1z+a_2z^2+\ldots).$$

Multiplying again both terms of the equation by $1-qz$, we find

$$\begin{aligned}1-(qz)^{\gamma} &= (1-qz)\big(1-\big(1+qz+\ldots+(qz)^{\gamma-1}\big)pz\big)\,(a_0+a_1z+a_2z^2+\ldots)\\ &= (1-z+pq^{\gamma}\cdot z^{\gamma+1})(a_0+a_1z+a_2z^2+\ldots)\\ &= a_0+\sum_{k=1}^{\gamma}(a_k-a_{k-1})z^k+\sum_{k=\gamma+1}^{\infty}(a_k-a_{k-1}+pq^{\gamma}\cdot a_{k-\gamma-1})z^k.\end{aligned}$$

Balancing the terms of degree 0 on both sides of the equation forces $a_0=1$. The terms of degree 1 to degree $\gamma-1$ are null on the left side, which implies $a_k-a_{k-1}=0$ and consequently $a_k=a_0=1$ for $k=1,2,\ldots,\gamma-1$. The term of degree γ is $-q^{\gamma}$ on the left side, so $a_{\gamma}-a_{\gamma-1}=-q^{\gamma}$, or $a_{\gamma}=1-q^{\gamma}$. Finally, all the other terms on the left side are null, which implies $a_k-a_{k-1}+pq^{\gamma}\cdot a_{k-\gamma-1}=0$, or $a_k=a_{k-1}-pq^{\gamma}\cdot a_{k-\gamma-1}$ for $k=\gamma+1,\gamma+2\ldots$

In conclusion, the Taylor coefficients $a_0, a_1, a_2,\ldots$ satisfy the recurrence equation

$$a_k=\begin{cases}1 & \text{if } k<\gamma,\\ 1-q^{\gamma} & \text{if } k=\gamma,\\ a_{k-1}-pq^{\gamma}\cdot a_{k-\gamma-1} & \text{otherwise.}\end{cases} \tag{3}$$

B.3 An alternative construction of $M_n(z)$

The alphabets described here are constructed so that there is exactly one way to decompose reads in symbols or in segments. For a given alphabet there exists only one transfer matrix that describes the reads that have no seed of minimum size γ. However, if we choose a different alphabet, we can obtain a different transfer matrix describing exactly the same reads.

In section 3.5, we decomposed the reads in the so-called skip-n alphabet $\mathcal{A}_n = \{\Box, |, \Downarrow_0, \Downarrow_1, \ldots, \Downarrow_n\}$, from which we derived the transfer matrix $M_n(z)$ shown in expression (6). We now introduce the alternative skip-n alphabet $\mathcal{A}_n^* = \{\Box, *, |, \Downarrow_0, \Downarrow_1, \ldots, \Downarrow_n\}$, where we add the symbol $*$.

The $*$ symbol stands for the nucleotides between a $\Downarrow_j$ terminator $(0 \le j \le n)$ and the following non-skipped position. Recall that seed can only start at non-skipped positions occurring every $n+1$ nucleotides, and that the $\Downarrow_j$ indicates a sequencing error j nucleotides before a non-skipped position. Figure 16 shows the read of Figure 7 in the alternative skip-3 alphabet. The $*$ symbols appear after the $\Downarrow_1$ and $\Downarrow_3$ terminators. They are not found after a $\Downarrow_0$ terminator because there are no nucleotides between this terminator and the following non-skipped position.

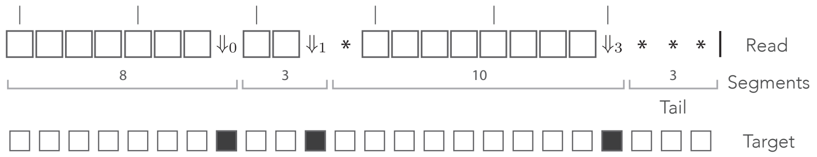

Figure 16: **The alternative skip encoding.** The read of Figure 7 is represented in the alternative skip-3 alphabet. The only difference is that the nucleotides between a $\Downarrow_j$ terminator $(0 \le j \le n)$ and the following non-skipped position are replaced by the $*$ symbol.

The match status of the $*$ nucleotides is irrelevant. Indeed, after a $\Downarrow_j$ symbol one has to "wait" until the next potential seed can start, j nucleotides further down. The weighted generating function of a $*$ nucleotide is thus z, as it stands for any nucleotide.

A sequence of $*$ symbols can end in only two ways: either with the end of the read (as in the case of Figure 16), or with a non-skipped position. If the end of the read comes first, the sequence of $*$ symbols is terminated by the usual $|$ terminator. Otherwise, the sequence has no terminator but it puts the read in exactly the same state as after a $\Downarrow_0$ terminator.

This means that in the alternative transfer matrix, the entries associated to $\Downarrow_j$ $(1 \le j \le n)$ simply "fast forward" to the entries associated with $\Downarrow_0$ and $|$. It takes exactly j nucleotides of any kind to go from a $\Downarrow_j$ symbol to the next non-skipped position, with weighted generating function z^j. If the end of the read occurs before the next non-skipped position, the $\Downarrow_j$ will be followed by a tail segment of up to $j-1$ symbols $*$, with weighted generating expression

$1+z+\ldots+z^{j-1}$. The segments following the $\Downarrow_0$ terminator are unchanged compared to the original expression of the transfer matrix.

Finally, the expression of the transfer matrix $M_n^*(z)$ in the alternative skip-n alphabet is

$$\begin{array}{c} \\ \Downarrow_0 \\ \Downarrow_1 \\ \vdots \\ \Downarrow_n \\ | \end{array}\begin{array}{c} \begin{array}{ccccc} \Downarrow_0 & \Downarrow_1 & \ldots & \Downarrow_n & | \end{array} \\ \begin{bmatrix} H_{0,0}(z) & H_{0,1}(z) & \ldots & H_{0,n}(z) & J_0(z) \\ z & 0 & \ldots & 0 & N_0(z) \\ \vdots & \vdots & \ddots & \vdots & \vdots \\ z^n & 0 & \ldots & 0 & N_{n-1}(z) \\ 0 & 0 & \ldots & 0 & 0 \end{bmatrix} \end{array}, \tag{29}$$

where p is the error rate of the sequencer, $q=1-p$, where n is the number of skipped nucleotides between potential seeds, where γ is the minimum seed length, and where

$$H_{0,j}(z)=(qz)^x\big(1+(qz)^{n+1}+\ldots+(qz)^{m(n+1)}\big)pz, \tag{4}$$

$$\text{with } x=-j-1 \pmod{n+1}, \text{ and } m=\left\lfloor\frac{\gamma-1-x}{n+1}\right\rfloor,$$

$$J_0(z)=1+qz+(qz)^2+\ldots+(qz)^{\gamma-1}, \tag{5}$$

$$N_i(z)=1+z+\ldots+z^i. \tag{12}$$

Observe that this expression is simpler than (6). It could have been used in section 3.5 because it is also easier to derive, but the burden of computations with expression (29) is heavier. The reason is that the top right entry of $M_n^*(z)^{s+1}$ is not the weighted generating function of reads with s sequencing errors ($s\geq 1$), so we cannot use the probability that the reads contains more than s errors as a bound on the neglected terms. As a consequence, we would need to perform more iterations in order to ascertain that the estimates are accurate to within chosen ε.

B.4 Proof that a_k is independent of $M_n(z)^m$ for $m>k+1$

In section 3.5 we considered the transfer matrix $M_n(z)$ of reads containing no skip-n seed. The quantity of interest a_k is the coefficent of z^k in the Taylor expansion of the top right term of $M_n(z)+M_n(z)^2+M_n(z)^3+\ldots$ We claimed that the coefficients $a_0,a_1,\ldots,a_k$ are independent of $M_n(z)^{k+2},M_n(z)^{k+3},\ldots$ so that they can be computed from the finite sum $M_n(z)+M_n(z)^2+\ldots+M_n(z)^{k+1}$.

We proceed by induction and show that for $k\geq 1$ all the entries of $M_n(z)^k$ are multiples of z^{k-1}. For $k=1$ the statement is trivial and there is nothing to show. Now assume that for some $k>1$, all the entries of $M_n(z)^k$ are multiples of z^{k-1}. Observe that from the definition of $M_n(z)$ in expression (6), the last row of $M_n(z)^m$ consists of zeros only for all $m\geq 1$. So in the matrix product $M_n(z)\cdot M_n(z)^k$, the polynomials $J_0(z),\ldots,J_n(z)$ in the last column of $M_n(z)$ are all multiplied by 0. The remaining terms of $M_n(z)$ are multiples of z, so

applying the induction hypothesis shows that all the terms of $M_n(z)^{k+1}$ are multiples of z^k.

Since all the terms of $M(z)^m$ are multiples of z^{k+1} for $m \geq k+1$, the coefficient of z^k is 0 in the top right term of every matrix $M(z)^m$ for $m \geq k+1$. As a result, the coefficient of z^k in the top right entry of $M_n(z)+M_n(z)^2+M_n(z)^3+\ldots$ is the same as in $M_n(z)+M_n(z)^2+\ldots+M_n(z)^{k+1}$, as we had to demonstrate. The same rationale holds for the coefficients of z^m $(0 \leq m < k)$, so finally the coefficients $a_0, a_1, \ldots, a_k$ can all be computed from the top right entry of $M_n(z)+M_n(z)^2+\ldots+M_n(z)^{k+1}$ as claimed.
